# The Origin of High Activity of Amorphous MoS_2_ in the Hydrogen Evolution Reaction

**DOI:** 10.1002/cssc.201901811

**Published:** 2019-08-08

**Authors:** Longfei Wu, Alessandro Longo, Nelson Y. Dzade, Akhil Sharma, Marco M. R. M. Hendrix, Ageeth A. Bol, Nora H. de Leeuw, Emiel J. M. Hensen, Jan P. Hofmann

**Affiliations:** ^1^ Laboratory for Inorganic Materials and Catalysis Department of Chemical Engineering and Chemistry Eindhoven University of Technology P.O. Box 513 5600 MB Eindhoven The Netherlands; ^2^ Netherlands Organization for Scientific Research (NWO) The European Synchrotron Radiation Facility (ESRF) CS40220 38043 Grenoble Cedex 9 France; ^3^ Faculty of Geosciences Utrecht University Princetonplein 9 3584 CC Utrecht The Netherlands; ^4^ School of Chemistry Cardiff University Main Building Park Place CF10 3AT Cardiff UK; ^5^ Department of Applied Physics Eindhoven University of Technology P.O. Box 513 5600 MB Eindhoven The Netherlands; ^6^ Laboratory of Physical Chemistry Department of Chemical Engineering and Chemistry Eindhoven University of Technology P.O. Box 513 5600 MB Eindhoven The Netherlands

**Keywords:** bond structure, electrocatalysis, molybdenum, operando spectroscopy, polymorphism

## Abstract

Molybdenum disulfide (MoS_2_) and related transition metal chalcogenides can replace expensive precious metal catalysts such as Pt for the hydrogen evolution reaction (HER). The relations between the nanoscale properties and HER activity of well‐controlled 2H and Li‐promoted 1T phases of MoS_2_, as well as an amorphous MoS_2_ phase, have been investigated and a detailed comparison is made on Mo−S and Mo−Mo bond analysis under operando HER conditions, which reveals a similar bond structure in 1T and amorphous MoS_2_ phases as a key feature in explaining their increased HER activity. Whereas the distinct bond structure in 1T phase MoS_2_ is caused by Li^+^ intercalation and disappears under harsh HER conditions, amorphous MoS_2_ maintains its intrinsic short Mo−Mo bond feature and, with that, its high HER activity. Quantum‐chemical calculations indicate similar electronic structures of small MoS_2_ clusters serving as models for amorphous MoS_2_ and the 1T phase MoS_2_, showing similar Gibbs free energies for hydrogen adsorption (Δ*G*_H*_) and metallic character.

## Graphical abstract

**The big short**: The presence of shorter Mo−Mo bonds in MoS_2_ is reported to enhance the electrocatalytic hydrogen evolution activity. Similar electronic and catalytic properties of 1T and amorphous MoS_2_, in contrast to 2H MoS_2_, are caused by shorter Mo−Mo and Mo−S bonds. This short Mo−Mo bond feature in 1T MoS_2_ is caused by Li intercalation and unstable under HER conditions, whereas it is intrinsic and stable for amorphous MoS_2_.

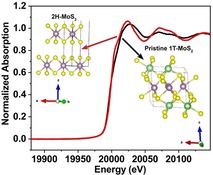

Scalable electrochemical proton reduction (hydrogen evolution reaction, HER) is crucial for realizing large‐scale storage of renewable energy. Water splitting requires efficient and robust catalysts, which are composed of earth‐abundant elements. The most active metal catalyst for HER is Pt, which is scarce and thus makes scale‐up of water electrolysis to terawatt (TW) scale too costly.^1^ Molybdenum disulfide (MoS_2_), one of the most studied transition metal chalcogenides (TMCs), has received substantial attention because of its unique physiochemical properties, such as a tunable band gap,^2^ high catalytic activity,^3^ and high electron mobility.^4^ These properties allow it to be exploited in transistors,^5^ metalloenzymes,^6^ and, at a practical scale, as the active phase in industrial catalysts for hydrotreatment of oil fractions.^7^ The ability to activate hydrogen reversibly also explains its promise for catalyzing hydrogen evolution in the context of electrochemical water splitting.^8^ Not surprisingly, the edge sites of MoS_2_ nanocatalysts have been identified as active HER sites by Jaramillo and co‐workers.^9^ Since then, tremendous efforts have been devoted to engineering the surface structure of MoS_2_ to preferentially expose these edge sites to improve HER performance.^10^

Different polymorphs of MoS_2_ exist in the form of 2H (trigonally coordinated), 1T (octahedrally coordinated), and 3R phases (rhombohedral).^3^, ^11^ 2H‐MoS_2_ is the thermodynamically stable two‐dimensional (2D) phase with semiconductor properties (band gap≈1.9 eV for monolayer, 1.2 eV for bulk),^12^ a low electron mobility, and a limited number of HER‐active (edge) sites. These properties render this phase less attractive for electrocatalytic applications^2^, ^13^ than the octahedral 1T phase, which is metallic and six orders of magnitude more conductive.13a The improved charge transfer kinetics and the affinity for binding H atoms on 1T‐MoS_2_ are reported to be responsible for the substantially enhanced HER activity compared to the 2H phase. However, the underlying mechanism of the high HER activity of the 1T phase has yet to be elucidated.^14^ As the 1T phase can be formed from 2H‐MoS_2_ by intercalation of cations (e.g., Li^+^, Na^+^),^15^ it is usually characterized by distorted structural domains.^16^ Aside from 2H and 1T phases, amorphous MoS_*x*_ (*x*=2–3) has also been extensively investigated in the past as a hydrotreatment catalyst and as a cathode material in lithium‐ion batteries.^17^ Furthermore, it was recently reported by Hu and other groups that this form of MoS_2_ is a highly active electrocatalyst for HER.8b, 18 Although Mo edge sites of 2H‐MoS_2_ have been experimentally identified as the active HER sites, the question of what causes the superior catalytic performances of 1T and amorphous phase MoS_2_ remains unclear, which adds to the challenge of unraveling the HER mechanism in amorphous MoS_2_.^11^, 17b, 17c, 19

Here, we show how the structure and surface properties of 2H, 1T, and amorphous MoS_2_ influence the HER activity and stability by a combined theory as well as ex situ and operando X‐ray spectroscopy approach. In comparison to 2H‐MoS_2_, shorter Mo−S and Mo−Mo bonds were observed in both 1T and amorphous MoS_2_ thin film electrodes. Besides, both core level Mo 3d and valence band photoemission spectra indicate that 1T and amorphous phase MoS_2_ exhibit a similar electronic structure. The short Mo−Mo bond in 1T phase MoS_2_ is caused by lithium intercalation and gradually changes back to the 2H phase accompanied by a decrease in HER activity at high overpotentials. By contrast, amorphous MoS_2_ (Am‐MoS_2_) retains its intrinsic (short) Mo−S and Mo−Mo bond structure as well as high HER activity after 24 h electrochemical testing under the same conditions. Electrochemical operando X‐ray absorption spectroscopy was performed to probe the local bond and electronic structure of MoS_2_ under HER conditions. The results show that the observed short Mo−Mo bonds play a key role in determining the activity of both 1T and amorphous phase MoS_2_ electrocatalysts for HER.

2H and amorphous MoS_2_ films were prepared by plasmaenhanced atomic layer deposition (PEALD) on glassy carbon plates at 450 and 250 °C, respectively, whereas the 1T phase was synthesized by lithium intercalation of the as‐deposited 2H‐MoS_2_ (see the Supporting Information for details). The HER electrocatalytic activity of as‐prepared MoS_2_ films was assessed in 0.1 m H_2_SO_4_ in a typical three‐electrode electrochemical cell. As shown in Figure 1, both cyclic voltammetry (CV) and linear sweep voltammetry (LSV) curves present higher current densities for 1T and amorphous MoS_2_, as compared to the 2H phase. However, even though 1T and amorphous MoS_2_ have comparable current densities initially, the catalytic activity of the 1T phase gradually decreases during the stability test, whereas Am‐MoS_2_ maintained its higher initial activity (Figure 1 d). This particular behavior led us to investigate further the electronic and structural properties of the materials.

**Figure 1:**
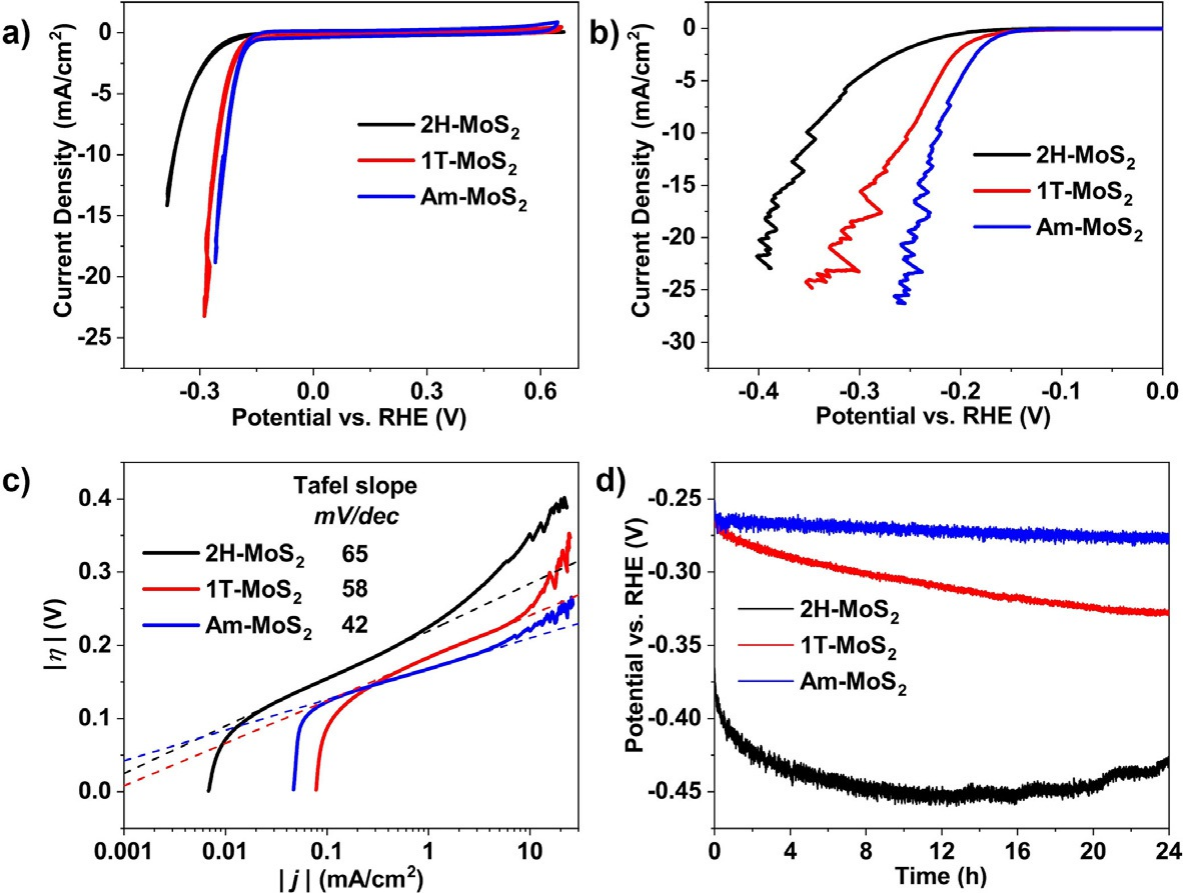
a, b) Cyclic voltammetry (CV; a) and linear sweep voltammetry (LSV; b) curves of 2H‐, 1T‐, and Am‐MoS_2_ films corrected by uncompensated resistance with scan rates of 50 mV s^−1^ for CV and 5 mV s^−1^ for LSV. c) Tafel slopes obtained from LSV curves in (b). d) Chronopotentiometric responses (*V*–*t*) recorded at a constant current density of 3 mA cm^−2^. Electrolyte: 0.1 m H_2_SO_4_.

We used X‐ray absorption spectroscopy at the Mo K‐edge to probe the electronic as well as local geometric structure of these films. Ex situ X‐ray absorption near‐edge spectra (XANES) of MoS_2_ films before and after HER stability tests recorded under a grazing incidence angle of 0.3° (grazing incidence X‐ray absorption spectroscopy)^23^ are shown in Figure 2. The suppression of features A and D in 1T (Figure 2 d) compared to 2H‐MoS_2_ emphasizes its distinct bond structure. Importantly, features A and D reappear for 1T‐MoS_2_ after 24 h HER stability testing, which implies that the 1T phase is not stable under the HER conditions and gradually changes back to 2H‐MoS_2_. Feature D is absent for the amorphous phase MoS_2_ both before and after the HER, indicating its stable bond structure (Figure 2 g), which is in contrast to 2H‐MoS_2_. Absorption edge features in the XANES spectra are very sensitive to the electronic properties of the atoms being probed:^20^ the less expressed shoulder at the edge and the shift of the white line for 1T‐MoS_2_ compared to 2H‐MoS_2_ are indicative of the structural differences. Simulations of the Mo‐K edge XANES spectra of MoS_2_ with hexagonal (2H phase) and monoclinic (1T phase with Li intercalation) symmetry were performed to understand these differences. The red curves (Figure 2 j, k) represent calculated spectra based on the model structure and the blue curves are calculated taking into account broadening by core‐hole lifetime effects.^21^ The fitted XANES spectra in both cases reproduce the experimental features of 2H‐ and 1T‐MoS_2_ well, which confirms their assignment. As monoclinic MoS_2_ shows octahedral Mo coordination with a shorter bond distance than 2H‐MoS_2_ upon Li intercalation, we may conclude that the as‐prepared 1T‐MoS_2_ in this study has a distorted bond structure. For further comparison, ex situ grazing incidence extended X‐ray fine structure (GI‐EXAFS) data of MoS_2_ films were recorded before and after stability measurements. The Fourier transform (FT) profiles in R‐space (Figure 2 b, c) present two main peaks at 2.40 Å and 3.16 Å (Table 1) corresponding to the nearest Mo−S and Mo−Mo bonds, respectively. The coordination number (CN) values shown in Table 1 suggest that there is no complete shell of S atoms around the central Mo at the surface of the MoS_2_ films, which can be due to termination by Mo edges or oxidation by emersion from the electrolyte and air exposure.^22^ By contrast, FT curves of 1T‐MoS_2_ exhibit a distinct decrease of the Mo−Mo bond length (short Mo−Mo bond) from 3.16 Å to 2.75 Å (Table 1), which corresponds to the characteristic bond length found in 1T phase MoS_2_.^24^, ^25^ Evidence for this feature can be also found by the larger Debye–Waller (*σ* ^2^) factor of both Mo−S and Mo−Mo bonds in 1T phase compared to 2H‐MoS_2_ (see the Supporting Information, Table S1) although the normal Mo−Mo bond (3.16 Å) is still present in 1T.^24^ Nevertheless, this shortened Mo−Mo bond disappeared after 24 h HER stability testing, which is consistent with the observations from XANES that 1T changes back to the 2H phase under these conditions. In the case of Am‐MoS_2_, a similarly short Mo−S and Mo−Mo bond structure was found (Figure 2 h, i). The similarities between the 1T and amorphous phases in XANES and EXAFS are also reflected in the Mo 3d core level and valence band photoemission spectra (Figure S3, Tables S2 and S3), shifting consistently to lower binding energies. Therefore, we may suggest that the short Mo−Mo bond features observed in both 1T‐MoS_2_ and Am‐MoS_2_ play a key role in enhancing the HER activity of MoS_2_ catalysts. Distinct from the 1T phase, the short Mo−Mo bond in Am‐MoS_2_ was retained after 24 h of HER stability testing. Considering the HER stability of Am‐MoS_2_, we may conclude that the bond structure in Am‐MoS_2_ is intrinsic (viz. not caused by Li intercalation), resulting in a higher stability during 24 h HER stability testing.

**Figure 2:**
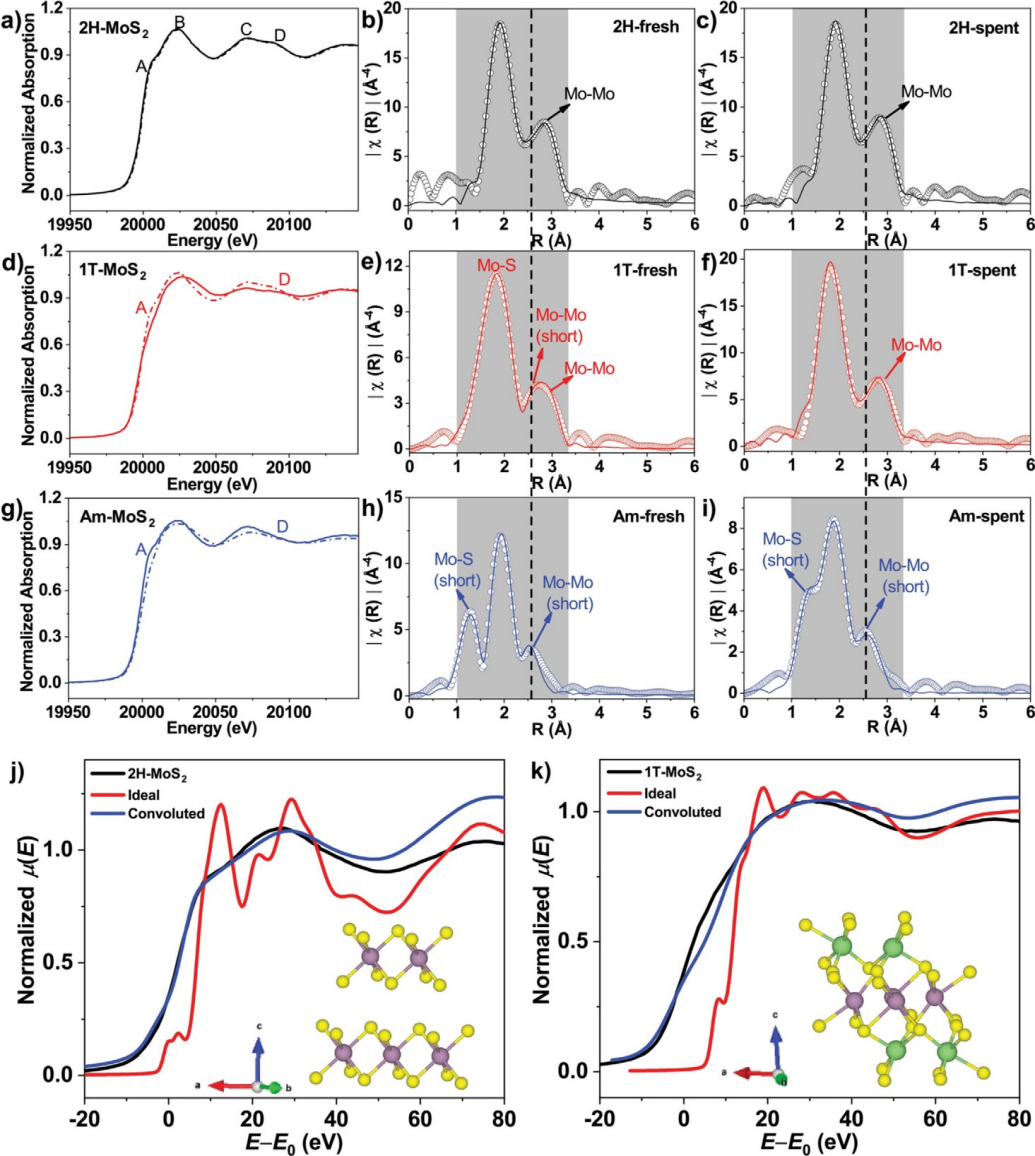
a, d, g) Mo K‐edge XANES spectra of 2H‐MoS_2_ (a), 1T‐MoS_2_ (d), and Am‐MoS_2_ (g) before (solid line) and after (dash line) stability test. b, e, h) Mo K‐edge Fourier transform EXAFS (*k* ^3^‐weighted) of 2H‐MoS_2_ (b), 1T‐MoS_2_ (e), and Am‐MoS_2_ (h) before stability test. c, f, i) Mo K‐edge Fourier transform EXAFS (*k* ^3^‐weighted) of 2H‐MoS_2_ (c), 1T‐MoS_2_ (f), and Am‐MoS_2_ (i) after stability test. j, k) Mo‐K edge XANES spectra of experimental data (black curve) and calculated simulation based on hexagonal (j, inset) and monoclinic (k, inset) structure model (purple, yellow, and green balls corresponds to Mo, S, and Li atoms, respectively); red curves represent simulated spectra whereas blue curves represent simulated spectra convoluted with the Mo 1s core‐hole lifetime.

**Table 1:** Summary of the ex situ grazing incidence Mo K‐edge EXAFS spectroscopic features obtained for MoS_2_ films under grazing incidence reflecting information about the top ≈3 nm of the material.^[a]^

Sample	Shell	Fresh		Spent	
		CN	*R* [Å]	CN	*R* [Å]
2H‐MoS_2_	Mo−S	4.25	2.402	4.80	2.405
	Mo−Mo	2.26	3.155	2.98	3.158
					
1T‐MoS_2_	Mo−S	3.06	2.419	5.86	2.365
	Mo−S (short)	1.78	2.019	–	–
	Mo−Mo	1.70	3.148	2.26	3.145
	Mo−Mo (short)	0.96	2.748	–	–
					
Am‐MoS_2_	Mo−S	5.29	2.430	3.50	2.368
	Mo−S (short)	0.60	1.767	0.56	1.802
	Mo−Mo (short)	1.08	2.778	1.57	2.824

[a] Detailed fitting parameters can be found in Table S1.

Several structural models for amorphous MoS_2_ or MoS_3_ have been proposed in previous reports by for example, Hibble et al.17b and Weber et al.^11^ However, based on our experimental observations, we cannot conclusively assign a structure to Am‐MoS_2_. Nonetheless, the disorder in amorphous MoS_2_ reported in this work is consistent with earlier reports.17a, 17d It is worth noting that even though the HER activity of 1T‐MoS_2_ decreases gradually (Figure 1 d), the corresponding overpotential is still much lower than that of 2H‐MoS_2_, which we have recently attributed to the presence of remaining Li adsorbed on the 1T‐MoS_2_ even after loss of intercalated Li.18c Inductively coupled plasma optical emission spectroscopy (ICP‐OES) analysis of MoS_2_ films after stability tests confirms the presence of adsorbed Li on 1T‐MoS_2_ (Table S4), and the adsorption of Li on MoS_2_ was observed to promote the activity of MoS_2_‐catalyzed hydrogen evolution reaction in our recent work.18c

To follow the structural evolution of the different MoS_2_ catalysts under HER conditions, an operando electrochemical cell (Figure S5) was developed and applied for X‐ray absorption spectroscopy experiments. The operando EXAFS spectra of different MoS_2_ polymorphs in dry state and at set potentials of +0.3 V and −0.3 V versus RHE in 0.1 m H_2_SO_4_ are shown in Figure 3. Table 2 summarizes the EXAFS fitting results. It can be seen that, despite a small reduction in CN for the Mo−Mo shell, the Mo−Mo and Mo−S bond distances as well as the Mo‐S CN remained the same within the accuracy range, pointing at the overall structural stability of 2H‐MoS_2_ under HER conditions. For both 1T and Am‐MoS_2_, a shortened Mo−S bond could be identified as well. In contrast to the disappearance of short Mo−S and Mo−Mo bonds after the 24 h stability test (Figure 2), the operando EXAFS data of 1T‐MoS_2_ confirms the retention of short Mo−S bonds under various potentials. On the one hand, the operando XAS was not carried out at grazing incidence angle and therefore reflects mostly bulk film information. On the other hand, the operando XAS measurements were performed at −0.3 V vs. RHE with a current density of only −200 μA cm^−2^ (Figure S6), while the 24 hours stability tests were performed at −3 mA cm^−2^.

**Figure 3:**
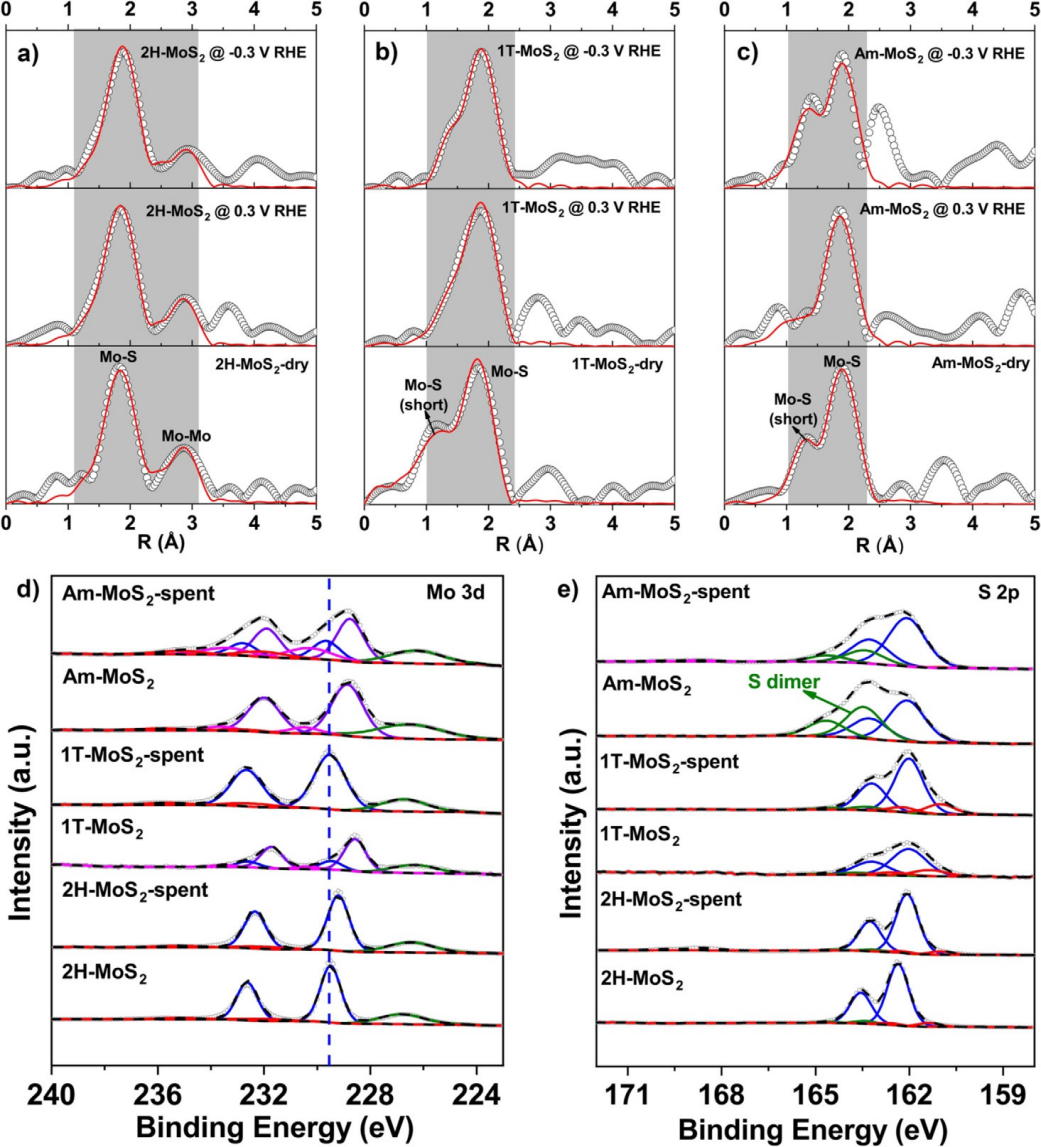
a–c) Mo K‐edge Fourier transform EXAFS (k^2^‐weighted) of 2H‐ (a), 1T‐ (b), and Am‐ (c) MoS_2_ under operando electrochemical conditions. Gray region represents R‐range for fitting. d, e) X‐ray photoemission spectra of Mo 3d (d) and S 2p (e) before and after (spent) operando XAS measurements.

**Table 2:** Summary of ex‐situ grazing incidence Mo K‐edge EXAFS spectroscopic features obtained for MoS_2_ films under operando HER conditions.^[a]^

Sample	Shell	Dry		+0.3 V		−0.3 V	
		CN	R [Å]	CN	R [Å]	CN	R [Å]
2H‐MoS_2_	Mo−S	4.28	2.385	4.33	2.391	4.71	2.403
	Mo−Mo	3.06	3.154	2.70	3.142	2.11	3.165
							
1T‐MoS_2_	Mo−S	6.66	2.354	5.82	2.416	3.78	2.406
	Mo−S (short)	5.90	2.004	0.96	1.835	0.34	1.827
							
Am‐MoS_2_	Mo−S	4.60	2.443	3.04	2.401	2.30	2.407
	Mo−S (short)	0.36	1.775	0.38	1.644	0.58	1.795

[a] Detailed fitting parameters can be found in Table S5, S6, and S7.

The surface electronic structures of 2H, 1T, and amorphous MoS_2_ films were probed by XPS before and after operando XAS measurements (Figure 3 d, e). The Mo 3d core level component describing the Mo−S bond for pristine 1T and Am‐MoS_2_ was shifted negatively by around 0.9 eV from that of the 2H phase, which is a characteristic feature for both 1T and amorphous phase MoS_2_.^18^, ^26^ However, the core level of Mo^IV^−S (Mo 3d_5/2_ 228.6 eV) for 1T‐MoS_2_ shifted back to 229.5 eV after operando XAS tests, suggesting a transformation from 1T to 2H‐MoS_2_ at the surface. By contrast, the shift in Mo 3d core level spectra for Am‐MoS_2_ (ca. 0.7 eV) remained unchanged after electrochemical tests (Figure 3 d). Raman spectroscopy was then used to further support the presence of different MoS_2_ phases (Figure S18).^16^ There is a redshift of E12g and A_1g_ peaks for 1T‐MoS_2_ compared to those of 2H‐MoS_2_, which stays constant before and after operando XAS measurements. The phase stability of the bulk 1T‐MoS_2_ film under mild reaction conditions is consistent with the EXAFS fitting results (Table 2). Even though sulfur dimers of Am‐MoS_2_ have been reported to be involved in proton reduction,19c the decreased intensity of sulfur dimers (Figure 3 e) here apparently did not influence the HER activity (Figure S6).

We utilized grazing incidence X‐ray diffraction (GIXRD) to inspect the materials before and after operando XAS measurements. In the diffraction patterns of MoS_2_ films (Figure S15), the diffraction peaks at 2*θ*=14.2° (0 0 2) and 33.3° (1 0 1) indicative of 2H‐MoS_2_ are absent in the pristine 1T phase MoS_2_ sample. However, the re‐appearance of (0 0 2) and (1 0 1) reflections for the spent 1T‐MoS_2_ sample suggests that the material gradually changes back to 2H‐MoS_2_. In addition, scanning electron microscopy (SEM) images (Figure S16) reveal obvious morphology changes for the 1T phase after HER tests. By contrast, neither Raman spectroscopy (Figure S18) nor GIXRD show any peaks before and after HER testing for Am‐MoS_2_. So far, we may conclude that even under mild HER conditions, the surface bond structure for the 1T phase would disappear and change back into the 2H phase, whereas amorphous MoS_2_ retains its intrinsic short Mo−Mo bond feature and with that its high HER activity.

By using density functional theory (DFT), we compared the (electronic) structures of Mo_3_S_9_ and Mo_6_S_17_ clusters as a motif for Am‐MoS_2_ with those of 2H‐MoS_2_ and 1T‐MoS_2_ in order to understand differences in the Gibbs free energy of hydrogen adsorption (Δ*G*_H*_), which is considered as a relevant descriptor for HER activity.13b, 27 The Mo−Mo and Mo−S bond distances found for the two small clusters correspond to those observed in 1T‐MoS_2_ and are shorter than those in 2H‐MoS_2_ (Figure 4 and Figures S21 and S22). Together with the structural data derived from EXAFS for our samples, this provides good grounds to hypothesize that Am‐MoS_2_ consists of small MoS_*x*_ clusters with an increased S/Mo ratio and shortened Mo−Mo and Mo−S bonds, similar to what is known for crystalline 1T‐MoS_2_. We then explored how these structures affect the HER performance for which we computed the Gibbs free energies of hydrogen adsorption (Δ*G*_H*_, structures see Figures S23–S27). For optimum HER activity, the value of Δ*G*_H*_ should be close to zero.^28^ For Mo_3_S_9_, a Δ*G*_H*_ value of −0.06 eV was computed, which is much more favorable than values of +2.13 eV and +0.78 eV for the basal planes of 2H‐MoS_2_ and Li‐stabilized 1T‐MoS_2_, respectively. As the hydrogen activation and formation on MoS_2_ is known to occur at the edge terminations,9a, 29 we also computed Δ*G*_H*_ for hydrogen adsorption on the Mo‐edges of 2H‐MoS_2_ (−0.23 eV) and 1T‐MoS_2_ (−0.10 eV). These data confirm that the edges of the distorted 1T‐MoS_2_ phase are the preferred sites for HER in comparison to the edges of 2H‐MoS_2_ and further suggest that small clusters also have a favorable Δ*G*_H*_. Figure 4 also gives the partial density of states (PDOS; Figure 4 d–g) of the two investigated clusters, 1T‐MoS_2_ and 2H‐MoS_2_. It can be immediately seen that, similar to 1T‐MoS_2_, the Mo_3_S_9_ and Mo_6_S_17_ cluster models exhibit metallic character with their Fermi level crossing the Mo 3d orbitals. In contrast, 2H‐MoS_2_ is a semiconductor with a band gap of 1.59 eV, which is consistent with valence band spectroscopy (Figures S12 and S13) and earlier theoretical predictions.^30^, ^31^ The adsorption of hydrogen does not induce significant changes to the electronic structures, although coupling is observed between H s orbital and Mo d and S p orbitals, consistent with weak bonding and high HER activity. The metallic nature of the Mo_3_S_9_ and Mo_6_S_17_ clusters and 1T‐MoS_2_ results in a higher intrinsic electronic conductivity for these materials than for the semiconducting 2H‐MoS_2_. Therefore, in addition to a more optimum free energy for hydrogen adsorption, the enhanced HER activity of 1T‐MoS_2_ and Am‐MoS_2_ can be further rationalized by a higher intrinsic electronic conductivity.

**Figure 4:**
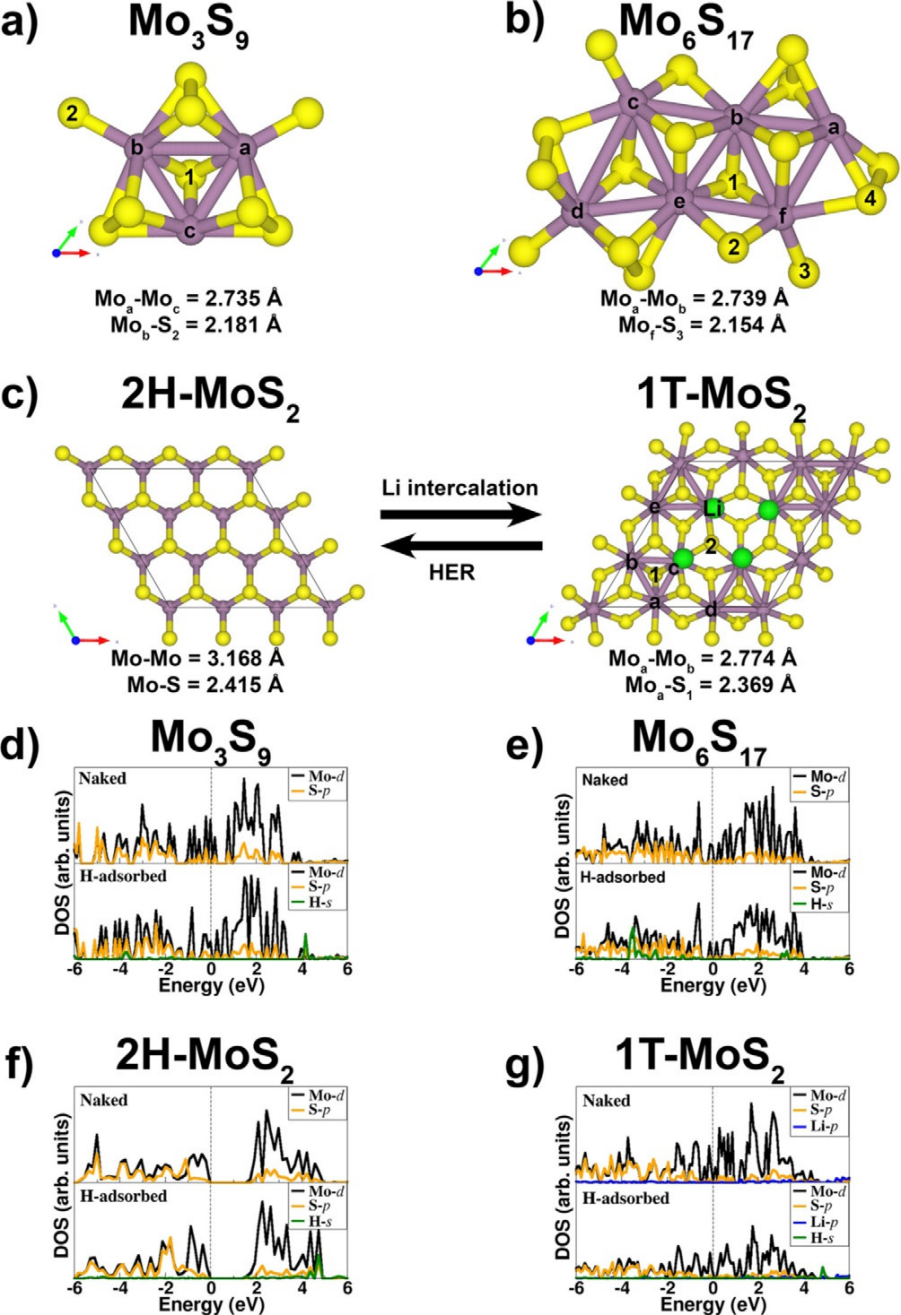
a, b) Optimized Mo_3_S_9_ (a) and Mo_6_S_17_ clusters serving as models for Am‐MoS_2_; c) Schematic illustration of the structural evolution between crystalline 2H and 1T MoS_2_ phases; normalized partial density of states (PDOS) of (d) Mo_3_S_9_, (e) Mo_6_S_17_ clusters, (f) 2H‐MoS_2_, and (g) 1T‐MoS_2_.

In summary, we have provided both experimental and theoretical evidence for the importance of the short Mo−Mo bond structures of 1T and amorphous MoS_2_ in comparison to crystalline 2H‐MoS_2_ for explaining the higher HER performance. Whereas crystalline 1T‐MoS_2_ stabilized by intercalated Li^+^ also displays high performance, Li ions were found to dissolve in the electrolyte during electrochemical testing, resulting in a slow transformation back to the 2H‐MoS_2_ phase and a concomitant decrease in HER activity. In contrast, amorphous MoS_2_ retains much of its high HER activity during prolonged operation.

## Conflict of interest

*The authors declare no conflict of interest*.

## Supporting information

As a service to our authors and readers, this journal provides supporting information supplied by the authors. Such materials are peer reviewed and may be re‐organized for online delivery, but are not copy‐edited or typeset. Technical support issues arising from supporting information (other than missing files) should be addressed to the authors.

Supplementary

## References

[1a] D. Kong , J. J. Cha , H. Wang , H. R. Lee , Y. Cui , Energy Environ. Sci. 2013, 6, 3553–3558;

[1b] J. Xie , J. Zhang , S. Li , F. Grote , X. Zhang , H. Zhang , R. Wang , Y. Lei , B. Pan , Y. Xie , J. Am. Chem. Soc. 2013, 135, 17881–17888; 24191645 10.1021/ja408329q

[2] Y. Gong , Z. Liu , A. R. Lupini , G. Shi , J. Lin , S. Najmaei , Z. Lin , A. L. Elias , A. Berkdemir , G. You , H. Terrones , M. Terrones , R. Vajtai , S. T. Pantelides , S. J. Pennycook , J. Lou , W. Zhou , P. M. Ajayan , Nano Lett. 2014, 14, 442–449. 24368045 10.1021/nl4032296

[3] Y. Yu , G. H. Nam , Q. He , X. J. Wu , K. Zhang , Z. Yang , J. Chen , Q. Ma , M. Zhao , Z. Liu , F. R. Ran , X. Wang , H. Li , X. Huang , B. Li , Q. Xiong , Q. Zhang , L. Gu , Y. Du , W. Huang , H. Zhang , Nat. Chem. 2018, 10, 638–643. 29610461 10.1038/s41557-018-0035-6

[4] K. K. Liu , W. Zhang , Y. H. Lee , Y. C. Lin , M. T. Chang , C. Y. Su , C. S. Chang , H. Li , Y. Shi , H. Zhang , C. S. Lai , L. J. Li , Nano Lett. 2012, 12, 1538–1544. 22369470 10.1021/nl2043612

[5] X. Wang , H. Feng , Y. Wu , L. Jiao , J. Am. Chem. Soc. 2013, 135, 5304–5307. 23489053 10.1021/ja4013485

[6] Y. Li , A. Yamaguchi , M. Yamamoto , K. Takai , R. Nakamura , J. Phys. Chem. C 2017, 121, 2154–2164.

[7a] L. van Haandel , E. J. M. Hensen , T. Weber , Catal. Today 2017, 292, 67–73;

[7b] L.-A. S. Carlos , G. Morales-Guio , X. Hu , Chem. Soc. Rev. 2014, 43, 6555–6569. 24626338 10.1039/c3cs60468c

[8a] J. D. Benck , T. R. Hellstern , J. Kibsgaard , P. Chakthranont , T. F. Jaramillo , ACS Catal. 2014, 4, 3957–3971;

[8b] M. L. Tang , D. C. Grauer , B. Lassalle-Kaiser , V. K. Yachandra , L. Amirav , J. R. Long , J. Yano , A. P. Alivisatos , Angew. Chem. Int. Ed. 2011, 50, 10203–10207; 10.1002/anie.201104412 21956994

[8c] Q. Ding , B. Song , P. Xu , S. Jin , Chem 2016, 1, 699–726.

[9a] T. F. Jaramillo , K. P. Jørgensen , J. Bonde , J. H. Nielsen , S. Horch , I. Chorkendorff , Science 2007, 317, 100–102; 17615351 10.1126/science.1141483

[9b] J. C. B. H. Tributsch , J. Electroanal. Chem. 1977, 81, 97–111.

[10a] J. Kibsgaard , Z. Chen , B. N. Reinecke , T. F. Jaramillo , Nat. Mater. 2012, 11, 963–969; 23042413 10.1038/nmat3439

[10b] D. Kong , H. Wang , J. J. Cha , M. Pasta , K. J. Koski , J. Yao , Y. Cui , Nano Lett. 2013, 13, 1341–1347; 23387444 10.1021/nl400258t

[10c] C. Tsai , H. Li , S. Park , J. Park , H. S. Han , J. K. Nørskov , X. Zheng , F. Abild-Pedersen , Nat. Commun. 2017, 8, 15113. 28429782 10.1038/ncomms15113 PMC5530599

[11] T. Weber , J. C. Muijsers , J. W. Niemantsverdriet , J. Phys. Chem. 1995, 99, 9194–9200.

[12] C. Backes , N. C. Berner , X. Chen , P. Lafargue , P. LaPlace , M. Freeley , G. S. Duesberg , J. N. Coleman , A. R. McDonald , Angew. Chem. Int. Ed. 2015, 54, 2638–2642; 10.1002/anie.201409412 25612324

[13a] D. Voiry , A. Goswami , R. Kappera , C. D. C. C. E. Silva , D. Kaplan , T. Fujita , M. Chen , T. Asefa , M. Chhowalla , Nat. Chem. 2015, 7, 45–49; 25515889 10.1038/nchem.2108

[13b] X. Geng , W. Sun , W. Wu , B. Chen , A. Al-Hilo , M. Benamara , H. Zhu , F. Watanabe , J. Cui , T. P. Chen , Nat. Commun. 2016, 7, 10672. 26861766 10.1038/ncomms10672 PMC4749985

[14a] Q. Tang , D.-e. Jiang , ACS Catal. 2016, 6, 4953–4961;

[14b] Y. Yin , J. Han , Y. Zhang , X. Zhang , P. Xu , Q. Yuan , L. Samad , X. Wang , Y. Wang , Z. Zhang , P. Zhang , X. Cao , B. Song , S. Jin , J. Am. Chem. Soc. 2016, 138, 7965–7972. 27269185 10.1021/jacs.6b03714

[15a] W. Chen , J. Gu , Q. Liu , R. Luo , L. Yao , B. Sun , W. Zhang , H. Su , B. Chen , P. Liu , D. Zhang , ACS Nano 2018, 12, 308–316; 29185710 10.1021/acsnano.7b06364

[15b] M. Acerce , D. Voiry , M. Chhowalla , Nat. Nanotechnol. 2015, 10, 313–318. 25799518 10.1038/nnano.2015.40

[16] S. J. R. Tan , S. Sarkar , X. Zhao , X. Luo , Y. Z. Luo , S. M. Poh , I. Abdelwahab , W. Zhou , T. Venkatesan , W. Chen , S. Y. Quek , K. P. Loh , ACS Nano 2018, 12, 5051–5058. 29709174 10.1021/acsnano.8b02649

[17a] S. P. Cramer , K. S. Liang , A. J. Jacobson , C. H. Chang , R. R. Chianelli , Inorg. Chem. 1984, 23, 1215–1221;

[17b] S. J. Hibble , D. A. Rice , D. M. Pickup , M. P. Beer , Inorg. Chem. 1995, 34, 5109–5113;

[17c] M. de Boer , A. J. van Dillen , D. C. Koningsberger , J. W. Geus , J. Phys. Chem. 1994, 98, 7862–7870;

[17d] J. Xie , Y. Xie , ChemCatChem 2015, 7, 2568–2580.

[18a] D. Merki , S. Fierro , H. Vrubel , X. Hu , Chem. Sci. 2011, 2, 1262–1267;

[18b] C. G. Morales-Guio , X. Hu , Acc. Chem. Res. 2014, 47, 2671–2681; 25065612 10.1021/ar5002022

[18c] L. Wu , N. Y. Dzade , M. Yu , B. Mezari , A. J. F. van Hoof , H. Friedrich , N. H. de Leeuw , E. J. M. Hensen , J. P. Hofmann , ACS Energy Lett. 2019, 4, 1733–1740. 31328171 10.1021/acsenergylett.9b00945 PMC6630958

[19a] S. J. Hibble , G. B. Wood , J. Am. Chem. Soc. 2004, 126, 959–965; 14733573 10.1021/ja037666o

[19b] R. I. Walton , A. J. Dent , S. J. Hibble , Chem. Mater. 1998, 10, 3737–3745;

[19c] B. Lassalle-Kaiser , D. Merki , H. Vrubel , S. Gul , V. K. Yachandra , X. Hu , J. Yano , J. Am. Chem. Soc. 2015, 137, 314–321; 25427231 10.1021/ja510328m PMC4304453

[19d] A. Sharma , M. A. Verheijen , L. Wu , S. Karwal , V. Vandalon , H. C. M. Knoops , R. S. Sundaram , J. P. Hofmann , W. M. M. Kessels , A. A. Bol , Nanoscale 2018, 10, 8615–8627. 29696289 10.1039/c8nr02339e

[20a] X. Li , H.-Y. Wang , H. Yang , W. Cai , S. Liu , B. Liu , Small Methods 2018, 2, 1700395;

[20b] N. Kornienko , J. Resasco , N. Becknell , C. M. Jiang , Y. S. Liu , K. Nie , X. Sun , J. Guo , S. R. Leone , P. Yang , J. Am. Chem. Soc. 2015, 137, 7448–7455. 26051104 10.1021/jacs.5b03545

[21] J. W. Liwen , F. Wan , B. R. Perdue , T. T. Fister , S. Kim , C. A. Apblettc , D. Prendergasta , Phys. Chem. Chem. Phys. 2016, 18, 17326–17329. 27314253 10.1039/c6cp02412b

[22] F. A. Lima , R. Bjornsson , T. Weyhermuller , P. Chandrasekaran , P. Glatzel , F. Neese , S. DeBeer , Phys. Chem. Chem. Phys. 2013, 15, 20911–20920. 24197060 10.1039/c3cp53133c

[23] J. Liu , R. E. Saw , Y. H. Kiang , J. Pharm. Sci. 2010, 99, 3807–3814. 20665844 10.1002/jps.22202

[24] Q. Liu , Q. Fang , W. Chu , Y. Wan , X. Li , W. Xu , M. Habib , S. Tao , Y. Zhou , D. Liu , T. Xiang , A. Khalil , X. Wu , M. Chhowalla , P. M. Ajayan , L. Song , Chem. Mater. 2017, 29, 4738–4744.

[25] K. E. Dungey , M. D. Curtis , J. E. Penner-Hahn , Chem. Mater. 1998, 10, 2152–2161.

[26] P. Afanasiev , H. Jobic , C. Lorentz , P. Leverd , N. Mastubayashi , L. Piccolo , M. Vrinat , J. Phys. Chem. C 2009, 113, 4139–4146.

[27] Z. Zeng , Z. Yin , X. Huang , H. Li , Q. He , G. Lu , F. Boey , H. Zhang , Angew. Chem. Int. Ed. 2011, 50, 11093–11097; 10.1002/anie.201106004 22021163

[28] P. D. Tran , T. V. Tran , M. Orio , S. Torelli , Q. D. Truong , K. Nayuki , Y. Sasaki , S. Y. Chiam , R. Yi , I. Honma , J. Barber , V. Artero , Nat. Mater. 2016, 15, 640–646. 26974410 10.1038/nmat4588 PMC5495159

[29a] J. K. Nørskov , T. Bligaard , A. Logadottir , J. R. Kitchin , J. G. Chen , S. Pandelov , U. Stimming , J. Electrochem. Soc. 2005, 152, J23–J26;

[29b] J. Greeley , T. F. Jaramillo , J. Bonde , I. B. Chorkendorff , J. K. Nørskov , Nat. Mater. 2006, 5, 909–913. 17041585 10.1038/nmat1752

[30a] M. R. Gao , M. K. Chan , Y. Sun , Nat. Commun. 2015, 6, 7493; 26138031 10.1038/ncomms8493 PMC4507019

[30b] X. Zhao , D. Fu , Z. Ding , Y. Y. Zhang , D. Wan , S. J. R. Tan , Z. Chen , K. Leng , J. Dan , W. Fu , D. Geng , P. Song , Y. Du , T. Venkatesan , S. T. Pantelides , S. J. Pennycook , W. Zhou , K. P. Loh , Nano Lett. 2018, 18, 482–490. 29253330 10.1021/acs.nanolett.7b04426

[31a] Q. Tang , D.-E. Jiang , Chem. Mater. 2015, 27, 3743–3748;

[31b] X. Guo , G. Yang , J. Zhang , X. Xu , AIP Adv. 2015, 5, 097174;

[31c] F. Xi , P. Bogdanoff , K. Harbauer , P. Plate , C. Höhn , J. Rappich , B. Wang , X. Han , R. van de Krol , S. Fiechter , ACS Catal. 2019, 9, 2368–2380.

