# Supplementary material for: The Origin of High Activity of Amorphous MoS2 in the Hydrogen Evolution Reaction
Source: ChemSusChem. 2019 Aug 8;12(19):4383–9. doi: 10.1002/cssc.201901811 (PMC6852468; doi:10.1002/cssc.201901811)
Supplement: Supplementary file 1 — Supplementary [file CSSC-12-4383-s001.pdf]

CHEMISTRY & SUSTAINABILITY
CHEMSUSCHEM
ENERGY & MATERIALS

# Supporting Information

## The Origin of High Activity of Amorphous $MoS_2$ in the Hydrogen Evolution Reaction

Longfei Wu,[a] Alessandro Longo,[b] Nelson Y. Dzade,[c, d] Akhil Sharma,[e] Marco M. R. M. Hendrix,[f] Ageeth A. Bol,[e] Nora H. de Leeuw,[c, d] Emiel J. M. Hensen,*[a] and Jan P. Hofmann*[a]

cssc_201901811_sm_miscellaneous_information.pdf

**Table of Contents**

**Part I: Experimental section** 4

**Part II: *Ex-situ* Grazing Incidence XANES, EXAFS, XPS, and ICP analysis** 7

Figure S1. Mo-K edge EXAFS spectra of $MoS_2$ films plotted as $\chi$ (k) with k-weight of 3 7

Table S1. Mo-K edge EXAFS fitting parameters of $MoS_2$ films 8

Figure S2. Mo-K edge XANES spectra of experimental data and simulations for 1T-$MoS_2$ 8

Figure S3. XPS spectrum for $MoS_2$ spent films 9

Table S2. Summary of Mo $3d_{5/2}$ binding energy for different 9

Table S3. Summary for the atomic ratio of sulfur species based on S 2p spectra 9

Table S4. ICP analysis results of pristine $MoS_2$ films and those after stability tests 10

Figure S4. EIS Nyquist plots for $MoS_2$ spent films 10

**Part III: *Operando* XANES and EXAFS** 11

Figure S5. Design of in-situ electrochemical cell 11

Figure S6. *In-situ* XANES spectra of $MoS_2$ films and recorded current density 12

Figure S7. XANES spectra comparison between *in-situ* and new spot 12

Figure S8. EXAFS spectra of 2H-$MoS_2$ films plotted as $\chi$ (k) with k-weight of 2 13

Table S5. EXAFS fitting results of 2H-$MoS_2$ films under in-situ conditions 13

Figure S9. EXAFS spectra of 1T-$MoS_2$ films plotted as $\chi$ (k) with k-weight of 2 14

Table S6. EXAFS fitting results of 1T-$MoS_2$ films under in-situ conditions 14

Figure S10. EXAFS spectra of Am-$MoS_2$ films plotted as $\chi$ (k) with k-weight of 2 15

Table S7. EXAFS fitting results of Am-$MoS_2$ films under in-situ conditions 15

**Part IV: Raman, SEM, XPS, and XRD analysis** 16

Figure S11. Li 1s for 1T-$MoS_2$ films before and after in-situ XAS measurements 16

Figure S12. Valence band spectra of 2H and 1T-$MoS_2$ 16

Figure S13. Valence band spectra of Am-$MoS_2$ 17

Table S8. Summary of Mo $3d_{5/2}$ binding energy in in-situ XAS samples 17

Table S9. Summary for the atomic ratio of sulfur species based on S 2p spectra 17

Figure S14. XPS of 2H, 1T and Am-$MoS_2$ after in-situ XAS measurements 18

Table S10. ICP analysis results of $MoS_2$ films after in-situ XAS tests 19

Figure S15. GI-XRD patterns of 2H-, 1T- and Am-$MoS_2$ 19

Figure S16. SEM images of 2H-, 1T- and Am-$MoS_2$ 20

Figure S17. HRTEM images of 2H- and Am-$MoS_2$ 20

Figure S18. Raman spectra of 2H-, 1T- and Am-$MoS_2$ 21

**Part V: Electrochemically Active Surface Area** 21

Figure S19. Electrochemical double layer capacitance ($C_{dl}$) measurements 22

Figure S20. $MoS_2$ Layer thickness evolution as a function of number of ALD cycles 23

Table S11. Summary of $C_{dl}$ values 23

**Part VI: Density Functional Theory (DFT) modelling** 23

Computational Details 23

Figure S21. Mo−Mo and Mo−S bond distances in (a) $Mo_3S_6$ and (b) $Mo_6S_{17}$ 24

Figure S22. Calculated interatomic Mo−Mo and Mo−S bond distances in 1T-$MoS_2$ 25

Figure S23. $\Delta G_{H*}$ for hydrogen adsorption on $Mo_3S_9$ cluster 25

Figure S24. $\Delta G_{H*}$ for hydrogen adsorption on $Mo_9S_{17}$ cluster 26

Figure S25. $\Delta G_{H*}$ for hydrogen adsorption on intermediate 2H-$MoS_2$, and 1T-$MoS_2$ 26

Figure S26. $\Delta G_{H*}$ for hydrogen adsorption on 2H-$MoS_2$ Mo-edge 27

Figure S27. $\Delta G_{H*}$ for hydrogen adsorption on 1T-$MoS_2$ Mo-edge 27

**References** 28

**Part I: Experimental Section**

***Plasma enhanced atomic layer deposition (PEALD) of $MoS_2$*****:** $MoS_2$ thin films were deposited at 250 (amorphous $MoS_2$) and 450 °C (2H-$MoS_2$) in an Oxford Instrument FlexALTM ALD reactor on glassy carbon substrates (Carbon-Vitreous-3000C-Foil-VC000550 bought from Goodfellow Cambridge Ltd. UK). The plasma enhanced ALD (PEALD) process was based on a combination of a metal organic precursor $[(N^tBu)_2(NMe_2)_2Mo]$ and $H_2S+H_2+Ar$ plasma as a co-reactant. The flow rate of $H_2S$, $H_2$ and Ar was fixed at 8, 2 and 40 ml/min, respectively. Further details of the PEALD process can be found in our previous work.[1]

***Preparation of 1T-$MoS_2$*****:** 1T-$MoS_2$ films were prepared via Li intercalation with butyl lithium (n-BuLi) (2.5 M in n-hexane, Sigma-Aldrich) as Li source. Briefly, 2H-$MoS_2$ films prepared by PEALD on glassy carbon plates were transferred in a dry glass bottle to a Ar filled glovebox, then 10 mL n-BuLi solution were added. Immersion was continued for 24 h. After the experiments, the films were washed with dry n-hexane for three times (the waste liquid was stored in a separate Schlenk bottle). The waste liquid containing n-BuLi was taken out of the glovebox and quenched first by dry isopropanol drop by drop in an ice bath. After waiting about 30 min, the waste was quenched by water (drop by drop) and disposed in the waste bin.

***X-ray absorption spectroscopy*** (XAS) was performed at BM26A (DUBBLE beamline) of ESRF, operating at 6 GeV with a beam current of 200 mA. Fresh samples and those after electrochemical stability tests were mounted in an $N_2$ protected sample holder, then measured at a grazing incidence angle of 0.3° in fluorescence detection mode. Each sample was recorded with 10 scans for X-ray Near Edge Spectroscopy (XANES) and Extended X-ray Absorption Fine Structure (EXAFS) spectroscopy respectively. Mo foil was used as a reference for energy calibration, all spectra were collected at Mo K-edge (20 keV). EXAFS

spectra were background subtracted and fitted with Viper.[2] Scattering paths were calculated by FEFF6 from molybdenite ($MoS_2$) crystal structure from American Mineralogist Crystal Structure Database.[3] The fitting range of Mo K-edge was $\Delta k$ = 2-11.8 Å$^{-1}$ and $\Delta R$ = 1.3-3.3 Å. Plotted spectra have a *k*-weight of 3 and were not phase-corrected.

***Operando* X-ray absorption spectroscopy (XAS)** was performed at BM26A (DUBBLE beamline) of ESRF, operating at 6 GeV with a beam current of 200 mA. X-ray Near Edge Spectroscopy (XANES) and Extended X-ray Absorption Fine Structure (EXAFS) spectroscopy were acquired under *operando* conditions (i.e. in electrolyte under potential control) in a home-designed (TU/e-EPC, Figure S5) electrochemical cell with flowing 0.1 M $H_2SO_4$ ($N_2$ saturated, flow rate 0.1 mL min$^{-1}$). The working electrode is a $MoS_2$ modified glassy carbon plate (22 × 22 × 2 mm$^3$), the counter electrode is Pt wire and the reference electrode a Leakless Miniature Ag/AgCl Reference Electrode (calibrated with respect to reversible hydrogen electrode (RHE): +0.251 V vs. RHE, Model ET072, eDAQ). Mo foil was used as a reference for energy calibration; all spectra were collected in fluorescence mode at the Mo K-edge (20 keV). EXAFS spectra were background subtracted and fitted with Viper. Scattering paths were calculated by FEFF6 from molybdenite ($MoS_2$) crystal structure from American Mineralogist Crystal Structure Database.[3b] The fitting range of Mo K-edge was $\Delta k$ = 2.00-11.06 Å$^{-1}$ and $\Delta R$ = 1.06-2.43 Å. Plotted spectra were not phase-corrected and have a *k*-weight of 2.

***Raman spectroscopy*** was performed on a Renishaw inVia Raman microscope equipped with a 514 nm laser, a grating with 1800 lines/mm, and a CCD detector. For each scan, 5 accumulations with acquisition time of 10 s were taken.

***TEM characterization*** was performed on a JEOL ARM 200F operated at 80 kV. For the top planar view images, $MoS_2$ films were grown on $SiN_x$ TEM windows, coated with ~5 nm ALD $SiO_2$. For the cross-sectional imaging, the $MoS_2$ film was coated with a $SiO_x$ film stack as a

protective layer and subsequently prepared using a standard FIB lift-out TEM sample preparation scheme.[1]

***X-ray photoelectron spectroscopy (XPS)*** was performed on a ThermoScientific K-Alpha instrument equipped with a monochromatic X-ray source ($E$(Al $K_{\alpha}$) = 1486.6 eV). Energy calibration was performed by using the C 1s peak of $sp^3$ carbon at 284.6 eV as a reference and the spectra were fitted by CasaXPS software.

***Scanning electron microscopy (SEM)*** images were obtained with a FEI Quanta 200 scanning electron microscope at an accelerating voltage of 5 kV.

***Grazing Incidence X-ray diffraction (GIXRD)*** was performed on a Bruker D8 Advance X-ray diffractometer in grazing incidence mode (0.3°) (Cu $K_{\alpha}$, $\lambda$ = 1.5419 Å) at 40 kV and 40 mA.

***Electrochemical tests*** were performed in a three-electrode electrochemical cell with Hg(I)/$Hg_2Cl_2$(s)/KCl (saturated) as reference electrode, Pt foil as counter electrode and the catalyst modified glassy carbon electrode (GCE) as working electrode. The reference electrode was calibrated with respect to reversible hydrogen electrode (RHE), and has a value of +0.269 V vs. RHE. HER measurements and other electrochemical characterization were carried out in Ar saturated 0.1 M $H_2SO_4$ if not mentioned otherwise. Cyclic voltammetry (CV) and linear sweep voltammetry (LSV) curves were recorded at scan rate of 50 and 5 mV $s^{-1}$, respectively. Electrochemical impedance spectroscopy (EIS) was carried out from 10 kHz to 1 Hz at open circuit potential (OCP) with an AC amplitude of 10 mV.

24 h HER stability tests were performed as chronopotentiometry at a current density of −3 mA/$cm^2$ in a three-electrode electrochemical cell with Hg(I)/$Hg_2Cl_2$(s)/KCl (saturated) as reference electrode, glassy carbon rods (Alfa Aesar, 6 mm in diameter, 100 mm in length) as counter electrode and the catalyst modified glassy carbon electrode (GCE) as working electrode. Electrolyte: Ar saturated 0.1 M $H_2SO_4$.

**Part II: *Ex-situ* Grazing Incidence EXAFS**

**XANES simulations:** In order to evaluate the different features observed in the XANES data, simulations have been performed using the FDMNES program package.[4] According to the code, simulated spectra are obtained via the multiple scattering theory in Green formalism based on the muffin-tin approximation on the potential shape.[4] In the simulated signals, the muffin-tin radii have been tuned to have a 10% overlap between the different spherical potentials. Moreover, to correct for inelastic losses, the Hedin-Lundqvist exchange potential has been used.[4] The approximation of non-excited absorbing atoms, which better reproduces the experimental data, has been adopted. The simulated XANES spectra were calculated considering that all the atoms are surrounding the absorber Mo within a 7 Å radius sphere.

In addition, in the simulation of the XANES spectra, the tabulated core-hole broadening together with an energy resolution of 1.9 eV have been set. The effect of the structural disorder has not been considered in the calculation of the theoretical signal. The XANES spectra have been energy calibrated and then were compared with the calculated spectra.

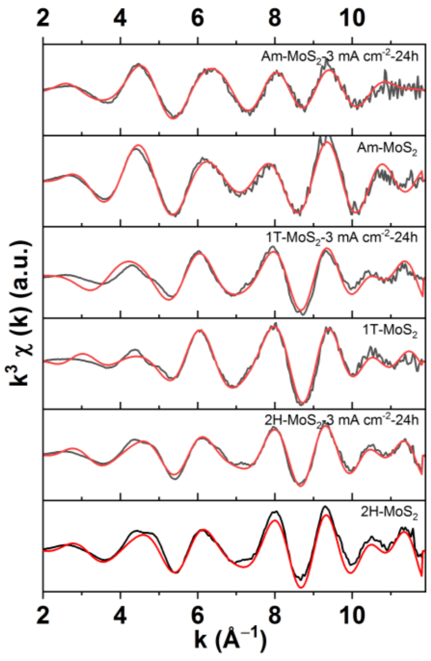


**Figure S1.** *Ex-situ* grazing incidence Mo-K edge EXAFS spectra of $MoS_2$ films plotted as χ (k) with k-weight of 3. The black curves represent experimental data and the red curves show fitted spectra.

**Table S1.** *Ex-situ* grazing incidence Mo-K edge EXAFS fitting parameters of $MoS_2$ films. [a] $\sigma^2$ was constrained during fitting. [b] R was constrained during fitting. [c] $\Delta E1$ and $\Delta E$ were set as floating parameters.

| **Sample** | **R factor (%)** | **k-range fit** | **R-range fit** | **Path** | **CN** | ***R* (Å)** | **$\sigma^2$ (Å$^2$)** | **$\Delta E$ (eV)** | **±CN** | **±*R* (Å) x $10^{-3}$** | **± $\sigma^2$ (Å$^2$) x $10^{-3}$** | **±$\Delta E$ (eV)** |
|---|---|---|---|---|---|---|---|---|---|---|---|---|
| **2H-$MoS_2$-fresh** | 2.96 | 2 - 11.82 | 1.46 – 3.16 | Mo-S | 4.25 | 2.402 | 0.002 | -0.91 | 0.01 | 0.68 | 0.064 | 0.05 |
| | 2.96 | 2 - 11.82 | 1.46 – 3.16 | Mo-Mo | 2.26 | 3.155 | 0.003 | -0.91 | 0.03 | 0.94 | -[a] | 0.05 |
| **2H- $MoS_2$@3 mA/cm$^2$-24h** | 2.76 | 2 - 11.82 | 1.46 – 3.16 | Mo-S | 4.80 | 2.405 | 0.003 | -1.03 | 0.01 | 0.05 | 0.001 | 0.01 |
| | 2.76 | 2 - 11.82 | 1.46 – 3.16 | Mo-Mo | 2.98 | 3.158 | 0.002 | -1.03 | 0.01 | 0.01 | 0.028 | 0.01 |
| **1T- $MoS_2$-fresh** | 4.71 | 2 - 11.82 | 1.30 – 3.32 | Mo-S | 3.06 | 2.419 | 0.005 | 1.76 | 0.27 | 3.11 | 0.843 | 0.22 |
| | 4.71 | 2 - 11.82 | 1.30 – 3.32 | Mo-S (short) | 1.78 | 2.019 | 0.006 | 1.76 | 0.28 | -[b] | 1.587 | 0.22 |
| | 4.71 | 2 - 11.82 | 1.30 – 3.32 | Mo-Mo | 1.70 | 3.148 | 0.004 | 1.76 | 0.51 | 5.74 | 1.939 | 0.22 |
| | 4.71 | 2 - 11.82 | 1.30 – 3.32 | Mo-Mo (short) | 0.96 | 2.748 | 0.015 | 1.76 | 0.50 | -[b] | -[a] | 0.22 |
| **1T- $MoS_2$@3 mA/cm$^2$-24h** | 7.12 | 2 - 11.82 | 1.50 – 3.00 | Mo-S | 5.86 | 2.365 | 0.004 | -12.30[c] | 1.15 | 8.68 | 1.632 | 1.61 |
| | 7.12 | 2 - 11.82 | 1.50 – 3.00 | Mo-Mo | 2.26 | 3.145 | 0.002 | -2.44[c] | 2.22 | 2.72 | 6.123 | 5.92 |
| **Am- $MoS_2$-fresh** | 6.68 | 2 - 11.82 | 1.00 – 3.00 | Mo-S | 5.29 | 2.430 | 0.001 | -0.06 | 0.04 | 0.58 | 0.208 | 0.0001 |
| | 6.68 | 2 - 11.82 | 1.00 – 3.00 | Mo-S (short) | 0.60 | 1.767 | 0.009 | -0.06 | 0.02 | 3.69 | 0.479 | 0.0001 |
| | 6.68 | 2 - 11.82 | 1.00 – 3.00 | Mo-Mo (short) | 1.08 | 2.778 | 0.003 | -0.06 | 0.01 | 7.28 | 0.553 | 0.0001 |
| **Am- $MoS_2$@3 mA/cm$^2$-24h** | 5.02 | 2 - 11.82 | 1.00 – 3.00 | Mo-S | 3.50 | 2.368 | 0.008 | -4.31 | 1.60 | 4.10 | 3.508 | 5.71 |
| | 5.02 | 2 - 11.82 | 1.00 – 3.00 | Mo-S (short) | 0.56 | 1.802 | 0.005 | -4.31 | 1.18 | 5.21 | 1.725 | 5.71 |
| | 5.02 | 2 - 11.82 | 1.00 – 3.00 | Mo-Mo (short) | 1.57 | 2.824 | 0.008 | 9.45 | 0.02 | 2.57 | 7.672 | 4.29 |

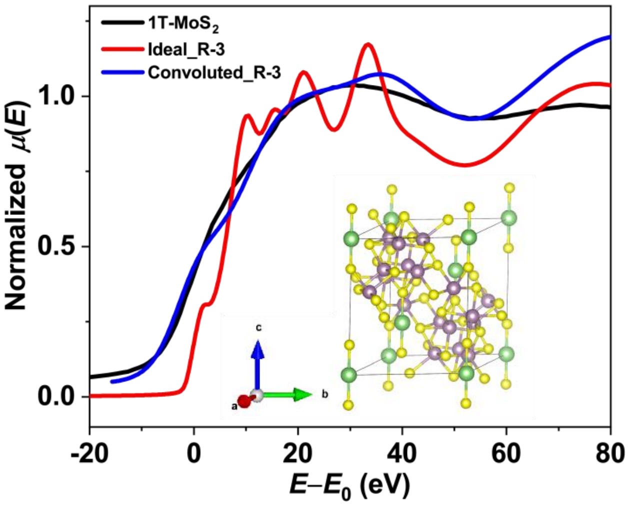


**Figure S2.** Mo K-edge XANES of 1T-$MoS_2$ (black curve) and calculated simulation (red, blue curve) based on rhombohedral structure (inset, purple, yellow and green balls corresponds to Mo, S and Li atoms respectively), the red curve represents the ideal simulated spectrum while the blue curve represents the simulated spectrum with consideration of broadening by core-hole lifetime.

Figure S2 presents the simulated Mo K-edge XANES spectra based on a rhombohedral $MoS_2$ structure upon Li intercalation. However, the calculated spectra differ from the experimental data in their main features, which means that the proposed rhombohedral structure is not

suitable. Therefore, it is assumed that the as-prepared 1T-$MoS_2$ has a monoclinic symmetry (Figure 2k) with distorted octahedral Mo coordination.

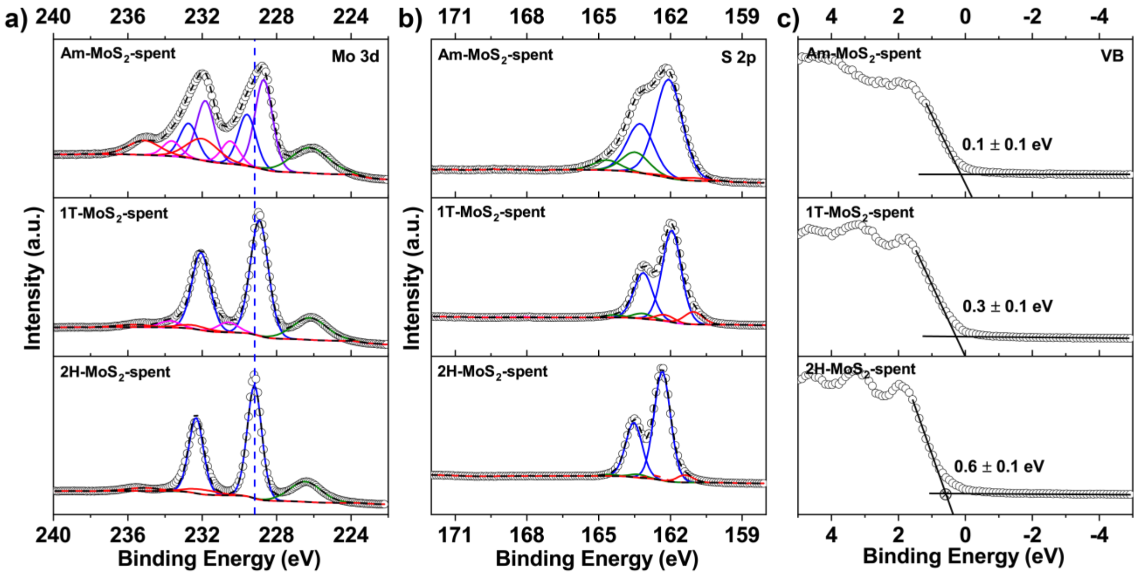


**Figure S3.** XPS spectrum of Mo 3d (a) and S 2p (b) for $MoS_2$ spent films; c) valence band spectra of corresponding samples, the Fermi edge energy was determined by the cross of two linear parts of the VB spectra.

**Table S2.** Summary of Mo(IV)-S (Mo $3d_{5/2}$) binding energies for different samples.

| | 2H-$MoS_2$ | 2H-$MoS_2$-spent | 1T-$MoS_2$ | 1T-$MoS_2$-spent | Am-$MoS_2$ | Am-$MoS_2$-spent |
|---|---|---|---|---|---|---|
| Mo $3d_{5/2}$ Binding Energy (eV) (Mo(IV)-S) | 229.5 | 229.2 | 228.6 | 228.9 | 228.8 | 228.7 |

**Table S3.** Summary for the atomic ratios of sulfur species based on S 2p spectra.

| % At. Conc. | 2H-$MoS_2$ | 2H-$MoS_2$-spent | 1T-$MoS_2$ | 1T-$MoS_2$-spent | Am-$MoS_2$ | Am-$MoS_2$-spent |
|---|---|---|---|---|---|---|
| Electron rich $S^{2-}$ | 5.6 | 6.2 | 9.6 | 6.0 | 1.1 | 2.4 |
| $S^{2-}$ | 90.0 | 91.1 | 83.3 | 88.7 | 56.0 | 81.1 |
| S in $S_2^{2-}$ | 4.4 | 2.7 | 7.1 | 5.1 | 42.9 | 16.5 |
| Sulfate | - | - | - | 0.2 | - | - |
| S/Mo ratio | 1.70 | 1.63 | 2.31 | 1.62 | 2.26 | 1.56 |

**Table S4.** ICP-OES analysis results of pristine $MoS_2$ films and those after 24 h HER stability tests.

| Sample name | Mo mass (μg) | S mass (μg) | Li mass (μg) | Formula (based on atomic ratio) |
|---|---|---|---|---|
| 2H-$MoS_2$-pristine | 172.0 (1.0)[a] | 141.6 (1.7)[a] | - | $MoS_{2.5}$ |
| 2H-$MoS_2$-spent | 122.6 (0.6)[a] | 130.4 (0.6)[a] | - | $MoS_{3.2}$ |
| 2H-$MoS_2$-spent solution | 0.3 (0)[a] | [b]- | - | - |
| 1T-$MoS_2$-pristine | 84.9 (0.2)[a] | 65.7 (0.4)[a] | 24.9 (-)[c] | $Li_{4.1}MoS_{2.3}$ |
| 1T-$MoS_2$-spent | 86.6 (0.3)[a] | 82.2 (1.0)[a] | 0.6 (-)[c] | $Li_{0.1}MoS_{2.8}$ |
| 1T-$MoS_2$-spent solution | 3.4 (0.1)[a] | [a]- | 9.8 (-)[c] | - |
| Am-$MoS_2$-pristine | 111.6 (0.6)[a] | 117.0 (1.8)[a] | - | $MoS_{3.1}$ |
| Am-$MoS_2$-spent | 54.0 (0.3)[a] | 70.9 (1.3)[a] | - | $MoS_{3.9}$ |
| Am-$MoS_2$-spent solution | 3.6 (0.1)[a] | [a]- | - | - |

[a]Standard deviations of the ICP values are calculated based on two emission lines of Mo.
[b]Sulfur quantification is not possible as the electrolyte has been 0.1 M $H_2SO_4$.
[c]Calculation of standard deviation is not possible as there is only one emission line of Li.

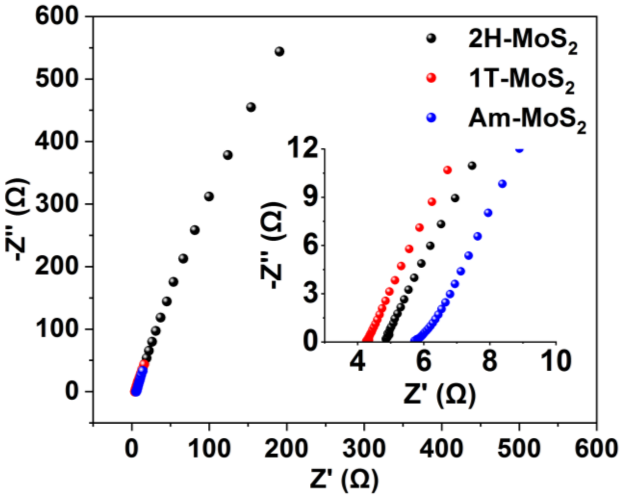


**Figure S4.** EIS Nyquist plots of corresponding films collected at open circuit potential ($V_{OCP}$) with an AC amplitude of 10 mV, the inset presents the zoom-in Nyquist plots.

**Part III:** ***Operando*** **XANES and EXAFS**

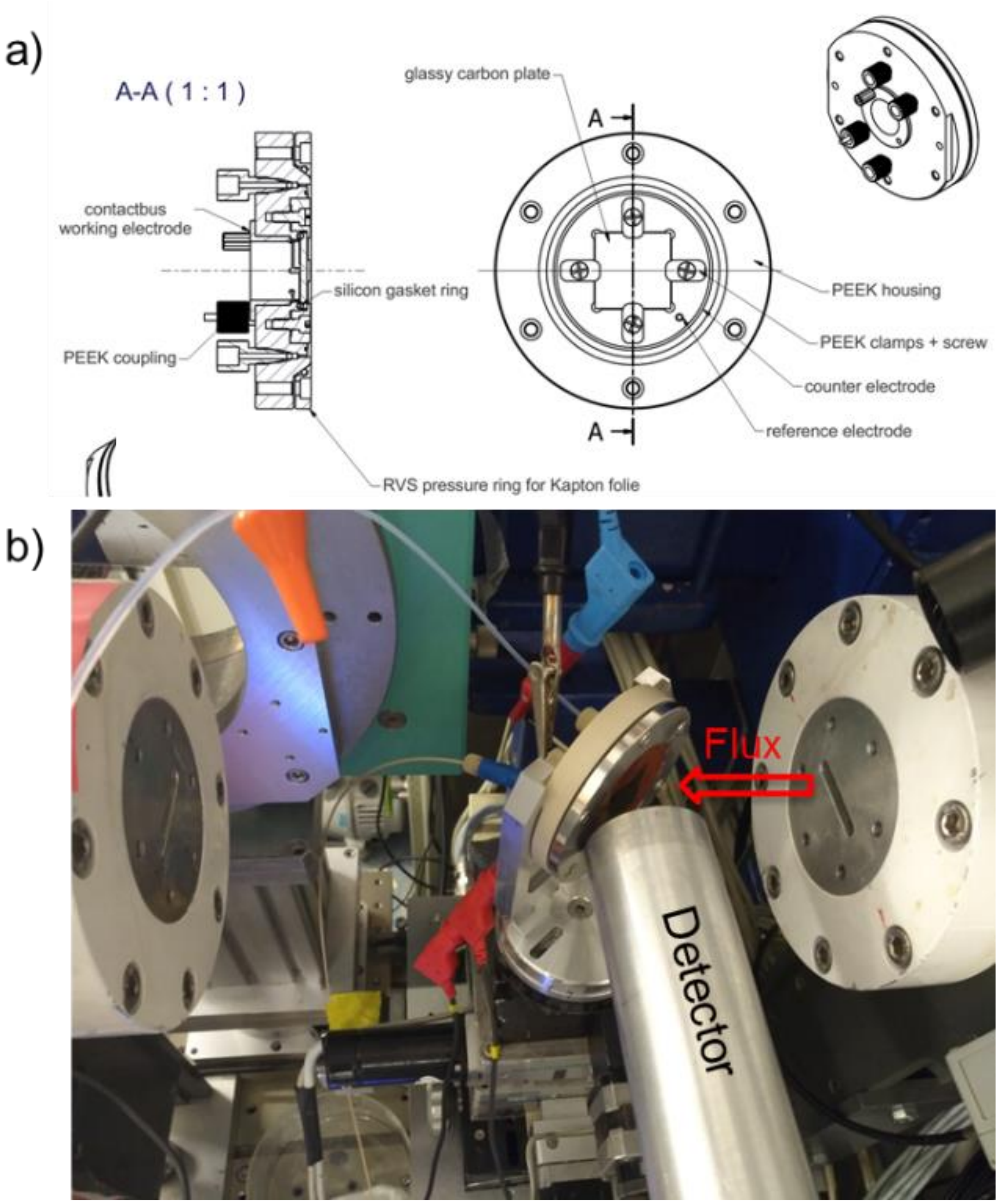


**Figure S5.** a) Design of the electrochemical cell for *operando* XAS measurements; b) photo of the electrochemical cell during experiments.

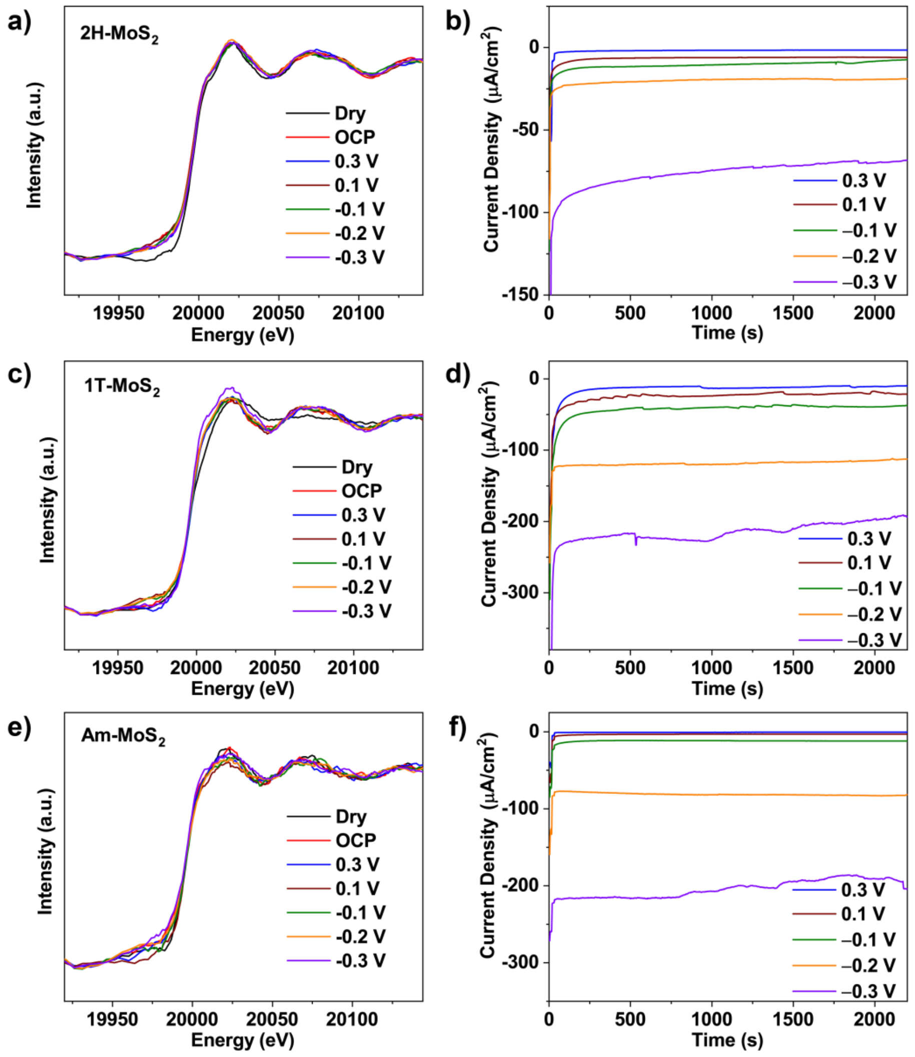


**Figure S6.** a, c, e) *Operando* XANES spectra of $MoS_2$ films under different conditions; b, d, f) Current density of 2H-$MoS_2$ (b), 1T-$MoS_2$ (d) and Am-$MoS_2$ (f) recorded during XANES measurements. Potentials are reported vs. RHE.

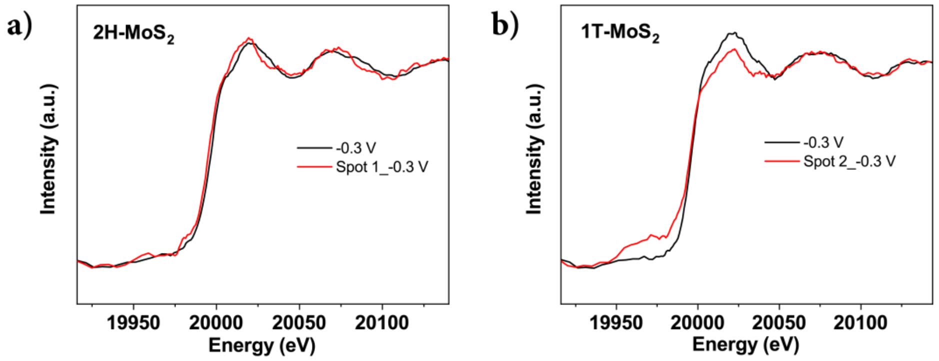


**Figure S7.** a, b) XANES spectra of $MoS_2$ films under *operando* condition and new spot with the same applied potential.

*Operando* XANES spectra shown in Figure S6 were plotted with a merge of 10 scans while the spectra for spot 1 and 2 in Figure S7 were plotted with only one quick scan. As shown in Figure S7, there is no obvious difference in XANES features between *in-situ* and spot 1, 2, which indicates that beam damage can be neglected.

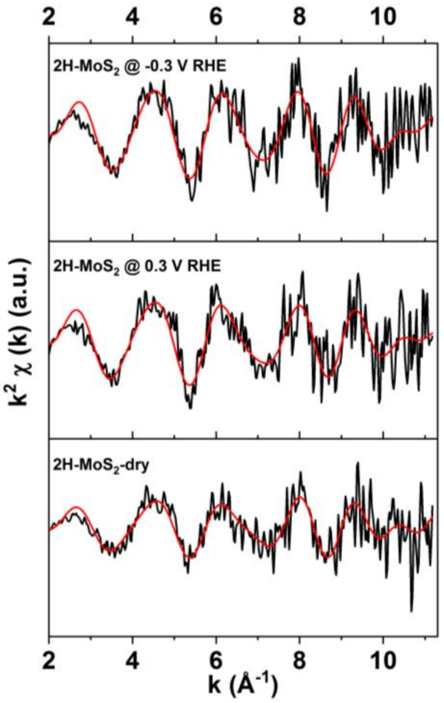


**Figure S8.** Mo-K edge EXAFS spectra of 2H-$MoS_2$ films plotted as χ (k) with k-weight of 2. The black curves represent experimental data and the red curves show fitted spectra.

**Table S5.** Mo-K edge EXAFS fitting results of 2H-$MoS_2$ films under *operando* conditions.

| Sample | R factor (%) | k-range fit | R-range fit | Path | CN | R (Å) | $\sigma^2$ (Å$^2$) | ΔE (eV) | ±CN | ±R (Å) | ± $\sigma^2$ (Å$^2$) x $10^{-3}$ | ±ΔE (eV) |
|---|---|---|---|---|---|---|---|---|---|---|---|---|
| 2H-$MoS_2$-dry | 13.5 | 2 - 11.18 | 1.08 – 3.08 | Mo-S | 4.28 | 2.385 | 0.003 | -3.20 | 0.09 | 0.003 | 0.093 | 0.17 |
| | 13.5 | 2 - 11.18 | 1.08 – 3.08 | Mo-Mo | 3.06 | 3.154 | 0.003 | -3.20 | 0.05 | 0.015 | 0.958 | 0.17 |
| 2H-$MoS_2$@0.3 V RHE | 8.1 | 2 – 11.18 | 1.08 – 3.08 | Mo-S | 4.33 | 2.391 | 0.004 | -3.06 | 0.04 | 0.001 | 0.084 | 0.01 |
| | 8.1 | 2 – 11.18 | 1.08 – 3.08 | Mo-Mo | 2.70 | 3.142 | 0.005 | -3.06 | 0.05 | 0.002 | 0.078 | 0.01 |
| 2H-$MoS_2$@−0.3 V RHE | 10.4 | 2 – 11.18 | 1.08 – 3.08 | Mo-S | 4.71 | 2.403 | 0.003 | -1.49 | 0.21 | 0.0004 | 0.009 | 0.48 |
| | 10.4 | 2 – 11.18 | 1.08 – 3.08 | Mo-Mo | 2.11 | 3.165 | 0.002 | -1.49 | 0.13 | 0.0006 | 0.133 | 0.48 |

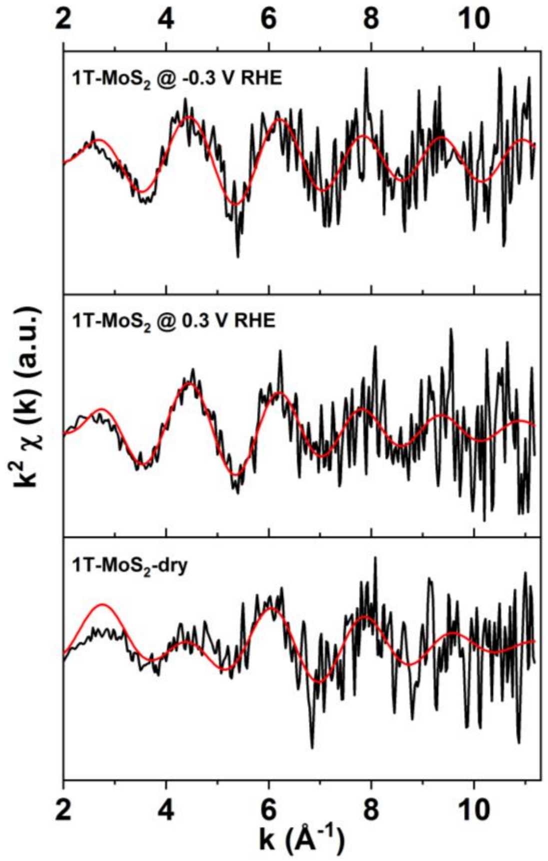


**Figure S9.** Mo-K edge EXAFS spectra of 1T-$MoS_2$ films plotted as χ (*k*) with *k*-weight of 2. The black curves represent experimental data and the red curves show fitted spectra.

As shown in Table S5, we assign the peak at ~2 Å to a short Mo-S bond which is very similar to Mo-O bond. However, XPS measurements indicate that molybdenum oxide species account for only 3.6 % of total Mo species at the surface of 1T-phase $MoS_2$. In addition, due to the configuration of the *operando* electrochemical cell, the collected EXAFS signals reflect bulk information of the samples. Therefore, it is more reasonable to assign this peak to short Mo-S bonds rather than to Mo-O bonds.

**Table S6.** Mo-K edge EXAFS fitting results of 1T-$MoS_2$ films under *operando* conditions.

| Sample | R factor (%) | k-range fit | R-range fit | Path | CN | R (Å) | $\sigma^2$ (Å$^2$) | ΔE (eV) | ±CN | ±R (Å) | ± $\sigma^2$ (Å$^2$) x $10^{-3}$ | ±ΔE (eV) |
|---|---|---|---|---|---|---|---|---|---|---|---|---|
| 1T-$MoS_2$-dry | 14.1 | 2 - 11.18 | 1.04 – 2.43 | Mo-S | 6.66 | 2.354 | 0.011 | -7.36 | 0.13 | 0.001 | 0.567 | 0.49 |
| | 14.1 | 2 - 11.18 | 1.04 – 2.43 | Mo-S (short) | 5.90 | 2.004 | 0.016 | -7.36 | 0.16 | 0.005 | 0.462 | 0.49 |
| 1T- $MoS_2$@0.3 V RHE | 8.4 | 2 – 11.18 | 1.04 – 2.43 | Mo-S | 5.82 | 2.416 | 0.007 | -6.00 | 0.07 | 0.002 | 0.137 | 0.21 |
| | 8.4 | 2 – 11.18 | 1.04 – 2.43 | Mo-S (short) | 0.96 | 1.835 | 0.014 | -6.00 | 0.13 | 0.005 | 0.454 | 0.21 |
| 1T- $MoS_2$@-0.3 V RHE | 5.0 | 2 – 11.18 | 1.04 – 2.43 | Mo-S | 3.78 | 2.406 | 0.003 | -2.05 | 0.01 | 0.0002 | 0.034 | 0.07 |
| | 5.0 | 2 – 11.18 | 1.04 – 2.43 | Mo-S (short) | 0.34 | 1.827 | 0.001 | -2.05 | 0.01 | 0.001 | 0.000 | 0.07 |

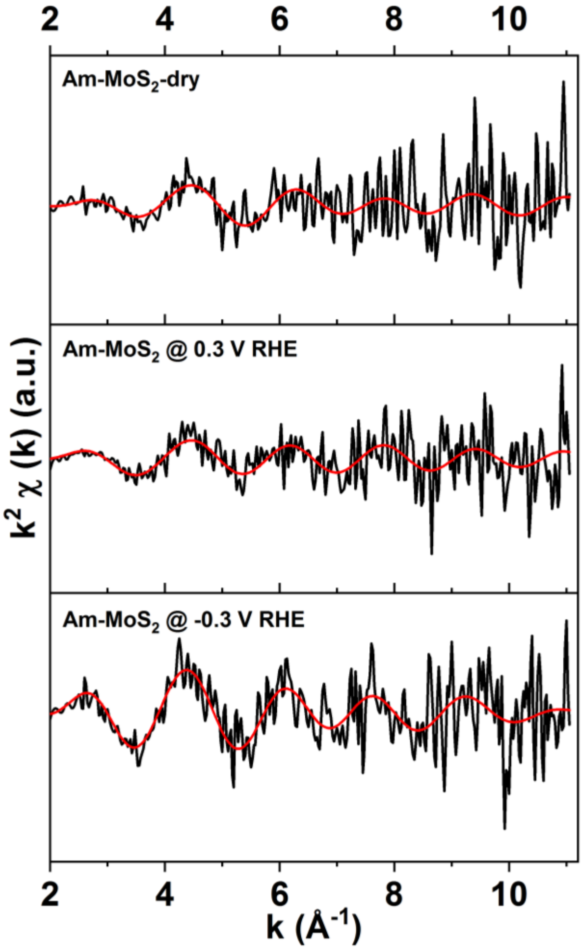


**Figure S10.** Mo-K edge EXAFS spectra of Am-$MoS_2$ films plotted as χ (k) with k-weight of 2. The black curves represent experimental data and the red curves show fitted spectra.

As shown in Table S6, we assign the peak at ~2 Å to a short Mo-S bond, which is very similar to a Mo-O bond. However, XPS measurements indicate that molybdenum oxide species account for 4.6 % of the total Mo species. In addition, due to the configuration of the *operando* electrochemical cell, the collected EXAFS signals reflect the bulk information of the samples. Therefore, it is more reasonable to assign this peak to short Mo-S bonds rather than to Mo-O bonds.

**Table S7.** Mo-K edge EXAFS fitting results of Am-$MoS_2$ films under *operando* conditions.

| Sample | R factor (%) | k-range fit | R-range fit | Path | CN | R (Å) | $\sigma^2$ (Å$^2$) | ΔE (eV) | ±CN | ±R (Å) | ± $\sigma^2$ (Å$^2$) x$10^{-3}$ | ±ΔE (eV) |
|---|---|---|---|---|---|---|---|---|---|---|---|---|
| Am-$MoS_2$-dry | 3.6 | 2 – 11.06 | 1.06 – 2.43 | Mo-S | 4.60 | 2.443 | 0.008 | -1.69 | 0.02 | 0.002 0.03 | 0.196 | 0.08 |
| | 3.6 | 2 – 11.06 | 1.06 – 2.43 | Mo-S (short) | 0.36 | 1.775 | 0.001 | -1.69 | 0.03 | 0.003 | 0.000 | 0.08 |
| Am-$MoS_2$@0.3 V RHE | 12.1 | 2 – 11.06 | 1.06 – 2.43 | Mo-S | 3.04 | 2.401 | 0.002 | -3.38 | 0.07 | 0.003 | 0.372 | 0.80 |
| | 12.1 | 2 – 11.06 | 1.06 – 2.43 | Mo-S (short) | 0.38 | 1.644 | 0.011 | -3.38 | 0.37 | 0.025 | 11.77 | 0.80 |
| Am-$MoS_2$@−0.3 V RHE | 14.2 | 2 – 11.06 | 1.06 -2.15 | Mo-S | 2.30 | 2.407 | 0.003 | -8.39 | 0.02 | 0.004 | 0.014 | 0.34 |
| | 14.2 | 2 – 11.06 | 1.06 -2.15 | Mo-S (short) | 0.58 | 1.795 | 0.003 | -8.39 | 0.12 | 0.009 | 0.000 | 0.34 |

## Part IV: Raman, SEM, and XPS analysis

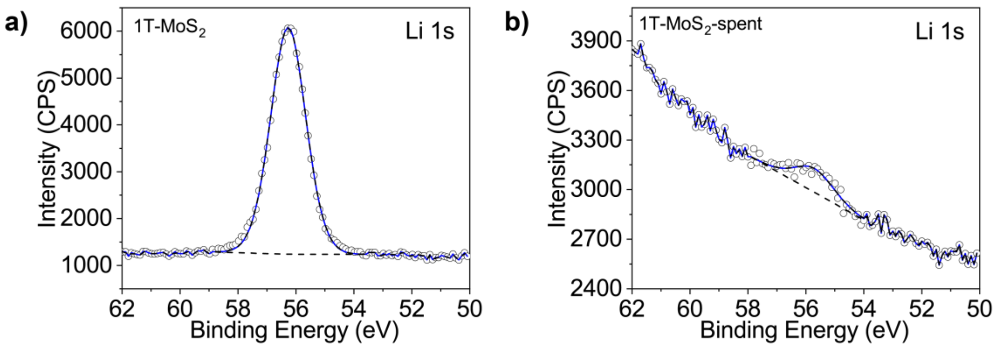


**Figure S11.** XPS spectrum of Li 1s for 1T-$MoS_2$ films before and after *operando* XAS measurements.

As shown in Figure S11, the surface lithium content was greatly reduced after *operando* XAS experiments, which means that lithium gradually leaches into the electrolyte during HER.

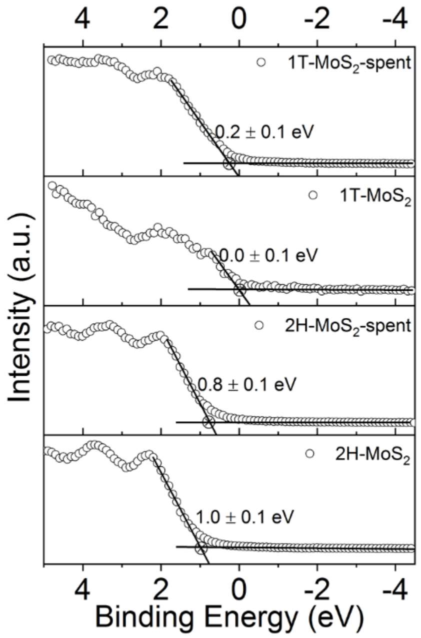


**Figure S12.** Valence band spectra of 2H and 1T-$MoS_2$ films. The Fermi edge energy was determined by the crossing of two linear parts of the VB spectra.

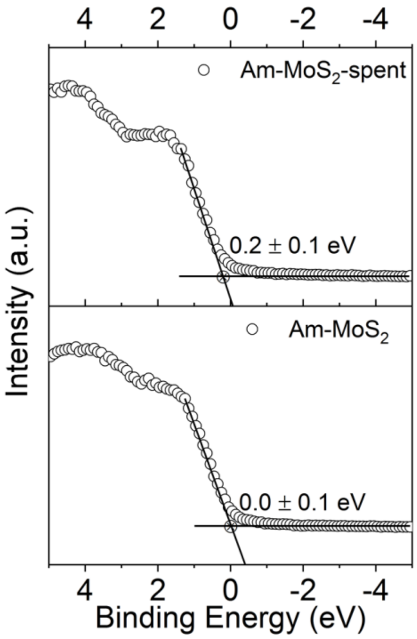


**Figure S13.** Valence band spectra and Fermi energy of Am-$MoS_2$ films. The Fermi edge energy was determined by the crossing of two linear parts of the VB spectra.

**Table S8.** Summary of Mo $3d_{5/2}$ binding energy for samples used in *operando* XAS measurements (corresponds to Figure 3d).

| | **2H-$MoS_2$** | **2H-$MoS_2$-spent** | **1T-$MoS_2$** | **1T-$MoS_2$-spent** | **Am-$MoS_2$** | **Am-$MoS_2$-spent** |
|---|---|---|---|---|---|---|
| Mo $3d_{5/2}$ Binding Energy (eV) (Mo(IV)-S) | 229.5 | 229.2 | 228.6 | 229.5 | 228.8 | 228.8 |

**Table S9.** Summary for the atomic percentage of sulfur species based on S 2p spectra (corresponds to Figure 3e).

| **% At. Conc.** | **2H-$MoS_2$** | **2H-$MoS_2$-spent** | **1T-$MoS_2$** | **1T-$MoS_2$-spent** | **Am-$MoS_2$** | **Am-$MoS_2$-spent** |
|---|---|---|---|---|---|---|
| Electron rich $S^{2-}$ | 5.6 | 2.6 | 9.6 | 14.8 | 1.1 | 1.1 |
| $S^{2-}$ | 90.0 | 92.1 | 83.3 | 79.8 | 56.0 | 76.2 |
| S in $S_2^{2-}$ (dimer) | 4.4 | 2.8 | 7.1 | 5.4 | 42.9 | 20.5 |
| Sulfate | - | 2.5 | - | - | - | 2.2 |
| S/Mo ratio | 1.70 | 1.73 | 2.31 | 1.63 | 2.26 | 1.68 |

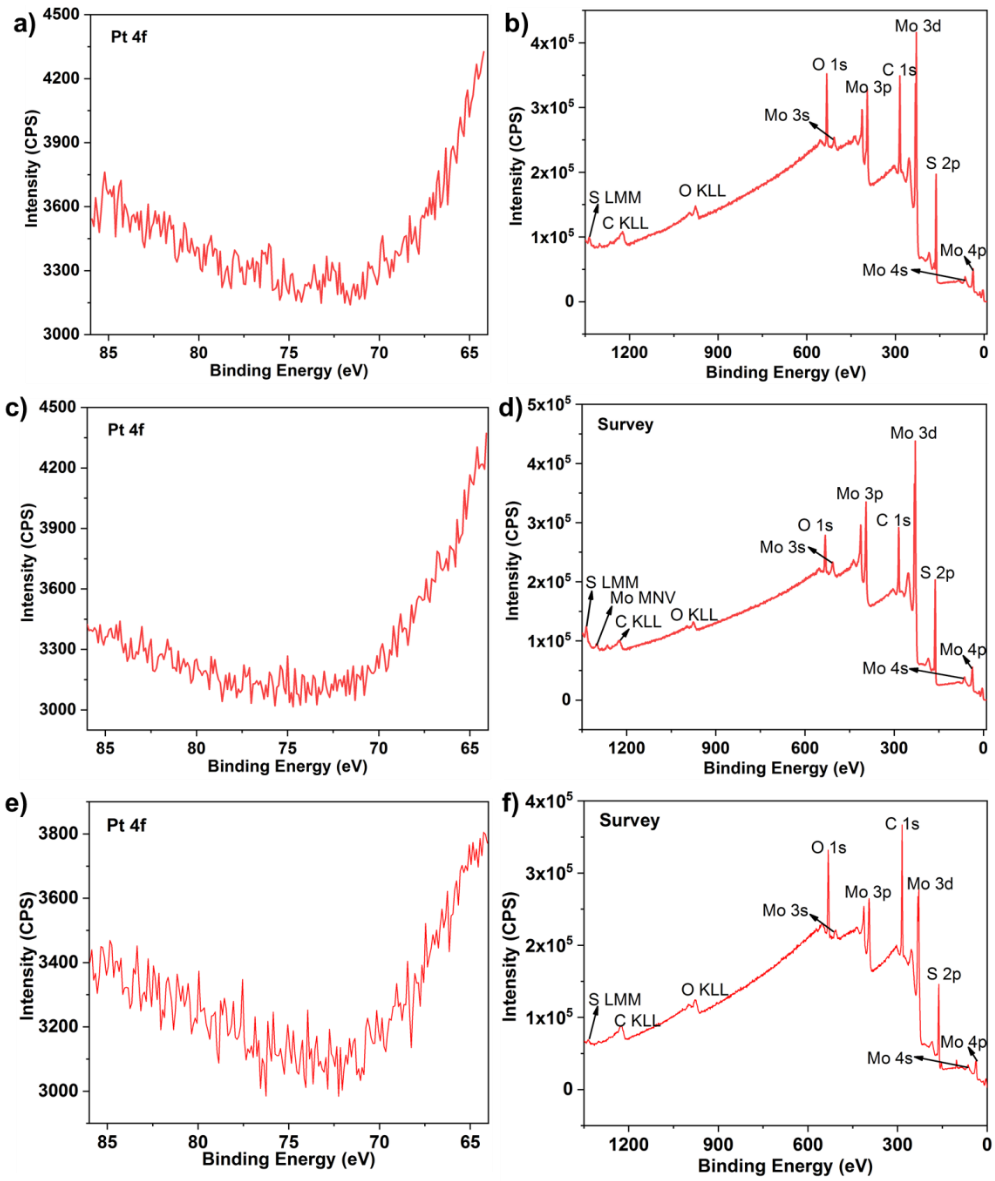


**Figure S14.** XPS spectrum of Pt 4f and survey for 2H- (a, b), 1T- (c, d) and Am- (e, f) $MoS_2$ films after *operando* XAS measurements.

As we use Pt wire as counter electrode in the *operando* electrochemical cell for XAS measurements, we performed XPS to check whether the films have been contaminated by Pt or not. Figure S14 shows the Pt 4f and survey spectra of 2H, 1T and amorphous $MoS_2$ films after *operando* XAS experiments. Pt contamination could not be observed in any of the samples.

**Table S10.** ICP analysis results of $MoS_2$ films after *operando* XAS experiments.

| Sample name | Mo mass (μg) | S mass (μg) | Li mass (μg) | Formula (based on atomic ratio) |
|---|---|---|---|---|
| 2H-$MoS_2$-spent | 170.1 (1.0)[a] | 146.7 (2.0)[a] | - | $MoS_{2.6}$ |
| 1T-$MoS_2$-spent | 83.9 (0.4)[a] | 78.4 (1.4)[a] | 4.5 (-) | $Li_{0.7}MoS_{2.8}$ |
| Am-$MoS_2$-spent | 24.4 (0.1)[a] | 28.2 (0.9)[a] | - | $MoS_{3.4}$ |

[a]Standard deviations are calculated on the ICP values of two emission lines of Mo.

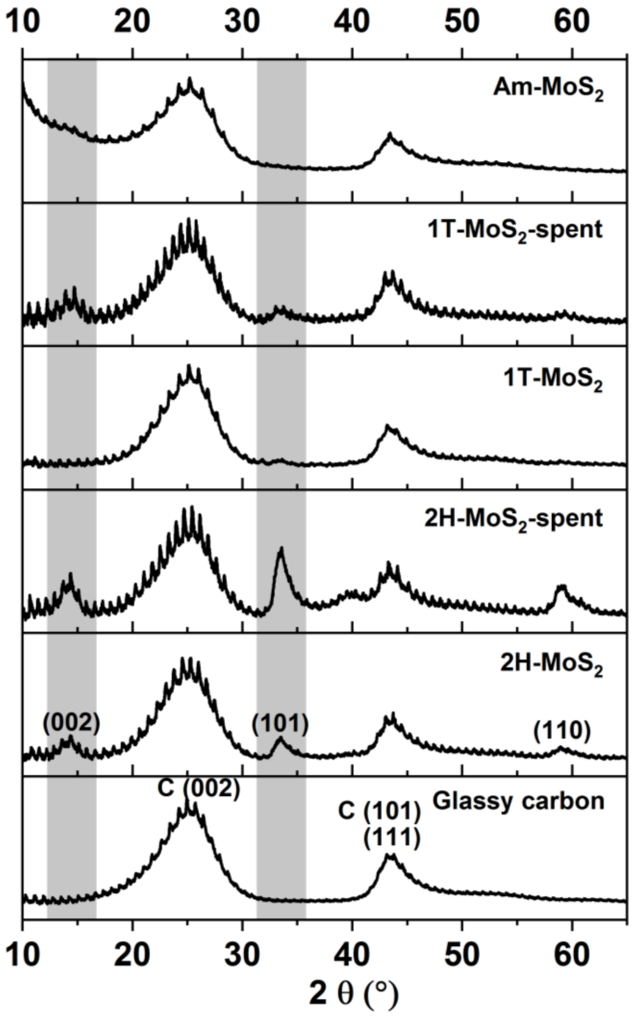


**Figure S15.** X-ray diffraction patterns with grazing incidence angle of 0.3° for $MoS_2$ films before and after *in-situ* XAS measurements.

The interference fringes, seen in all GIXRD patterns, can be ascribed to the glassy carbon substrate structure as shown in Figure S15.[8]

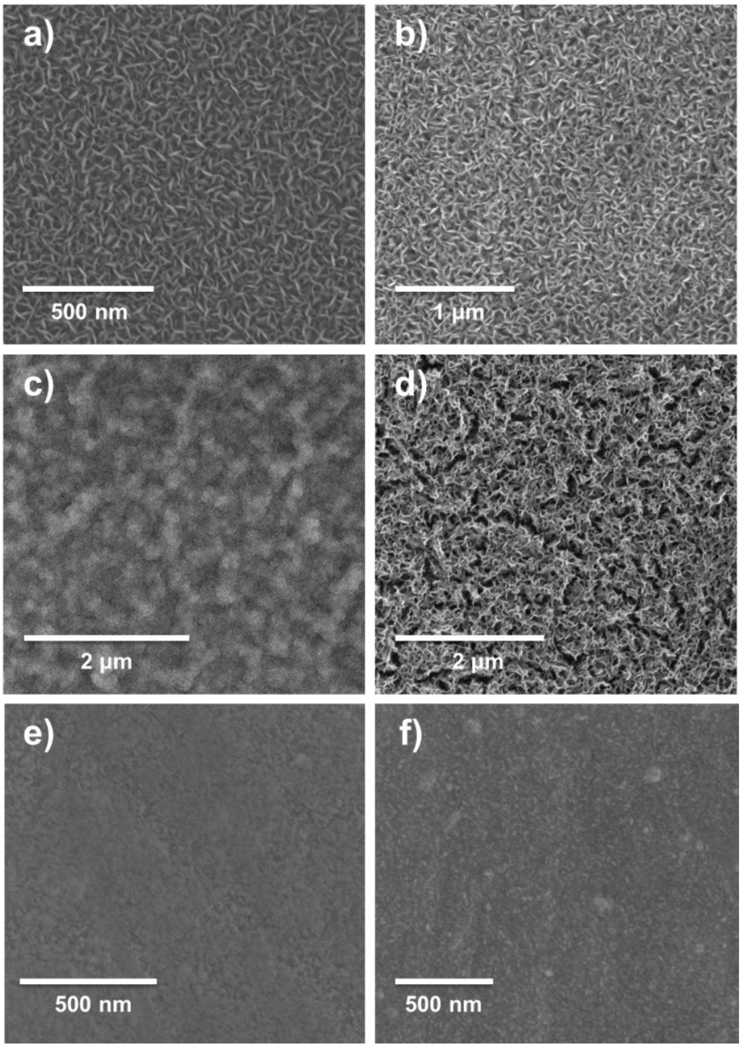


**Figure S16.** SEM images of 2H- (a, b), 1T- (c, d) and Am- (e, f) $MoS_2$ films before (a, c, e) and after (b, d, f) *operando* XAS measurements.

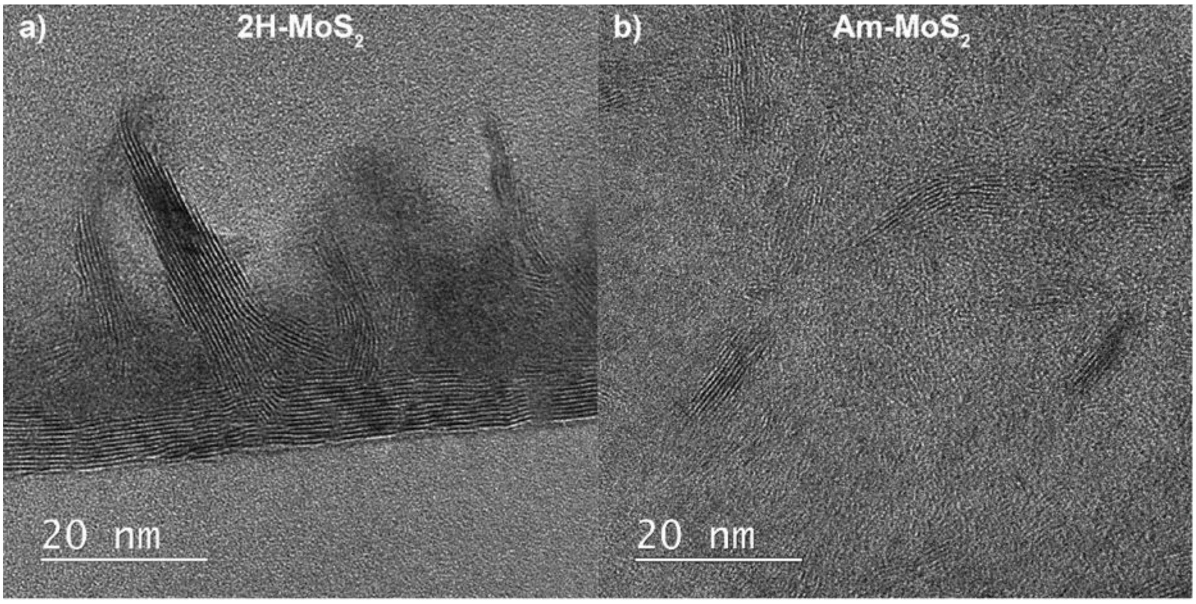


**Figure S17.** HRTEM images of 2H-$MoS_2$ (a, cross-section) and Am-$MoS_2$ (b). Figure S17a) is reproduced by permission of the Royal Society of Chemistry (RSC) from [1].

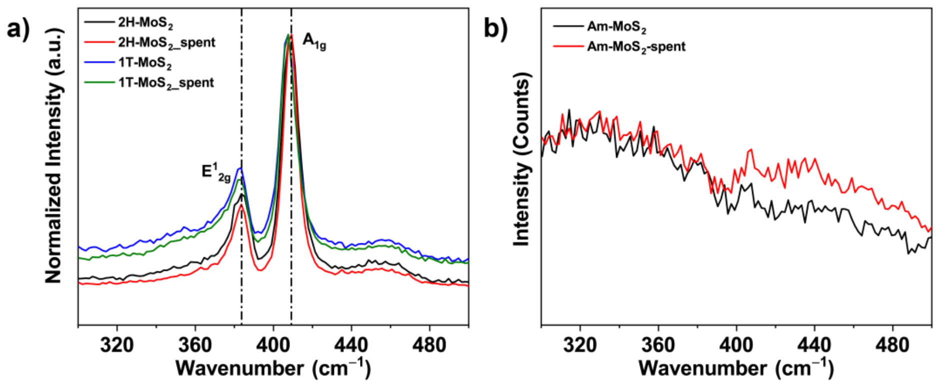


**Figure S18.** Raman spectra of 2H, 1T-$MoS_2$ (a, normalized with $A_{1g}$ peak) and Am-$MoS_2$ (b).

There exists a redshift of ~2 $cm^{-1}$ for $E^1_{2g}$ and $A_{1g}$ peaks of 1T-$MoS_2$ compared to 2H-$MoS_2$, which is characteristic for 1T-$MoS_2$, and the shift stays constant before and after *operando* XAS measurements.[5]

**Part V: Electrochemically Active Surface Area**

Typically, the electrochemical double layer capacitance ($C_{dl}$) method is applied to assess the electrochemically active surface area (ECSA). However, the ECSA value is obtained by the formula: $ECSA = \frac{C_{dl\,(sample)}}{C_{dl\,(single\,crystal)}}$, where $C_{dl(sample)}$ represents the $C_{dl}$ value of the sample and $C_{dl(single\,crystal)}$ represents the $C_{dl}$ of a single crystal reference material with certain orientation. This method assumes that the sample has the same orientation as the single crystal reference which is often not the case, and even single crystals with different orientations can have different $C_{dl}$ values.[6] Therefore, this method sometimes can over- or underestimate the real ECSA. For instance, as has been shown in Figure S19 and Table S11, 1T-$MoS_2$ has much higher $C_{dl}$ values than that of Am-$MoS_2$, which are comparable to their corresponding HER activities. In our case, since the $MoS_2$ polymorphs differ in material properties such as orientation, (electronic) structure and coordination, it would be inappropriate to use a single $C_{dl}$ value to compare their ECSA values. Considering that all $MoS_2$ samples were prepared with the same number of ALD cycles on glassy carbon substrates – the layer thickness as a function of ALD

cycles are shown in Figure S20 – we compare the electrocatalytic activity based on geometric surface area.

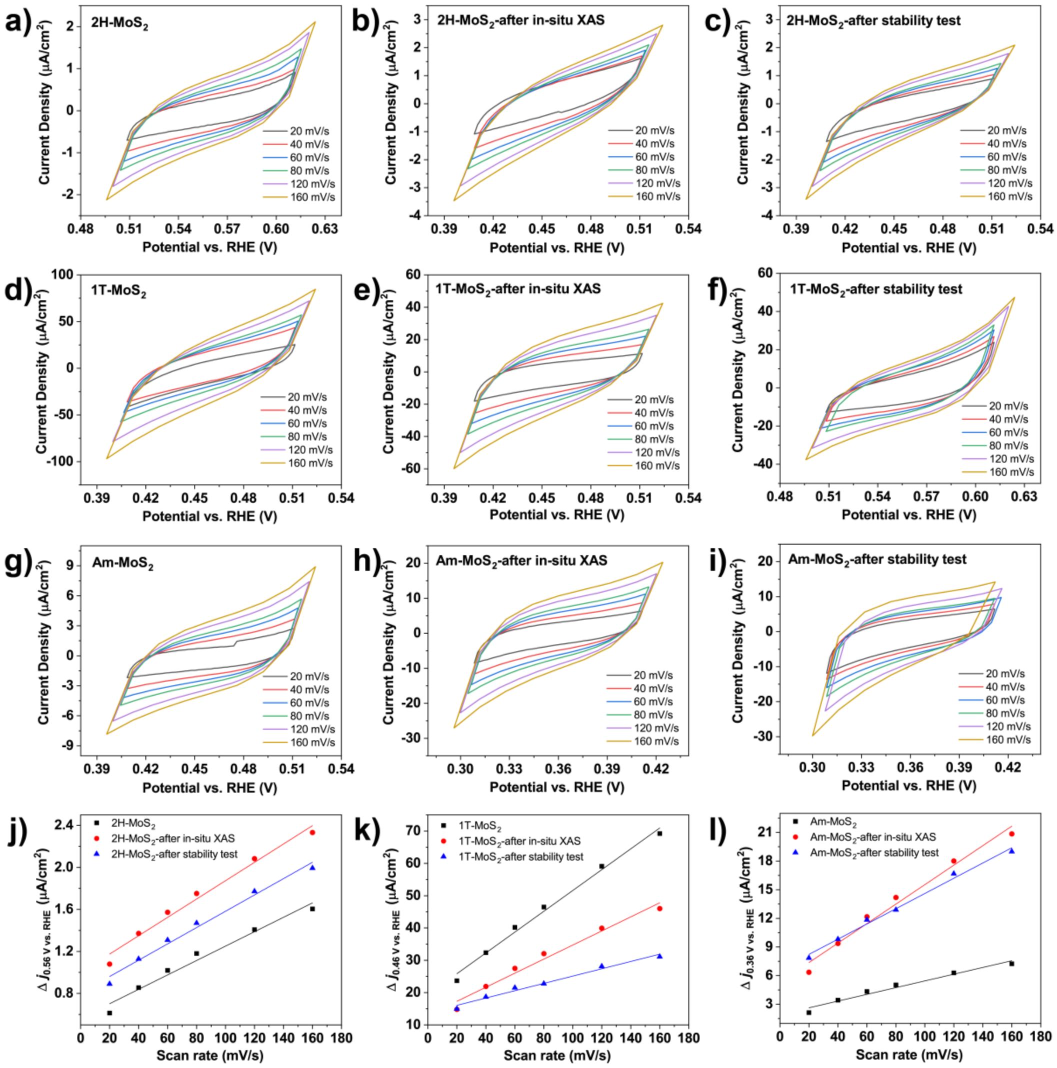


**Figure S19.** Electrochemical double layer capacitance ($C_{dl}$) measurements for $MoS_2$ films with a geometric surface area of 3.14 $cm^2$. a-i) Cyclic voltammetry (CV) measurements in the potential range of $V_{OCP}$ (open circuit potential) ± 50 mV; j-l) Fitting plots showing the extraction of corresponding $C_{dl}$.

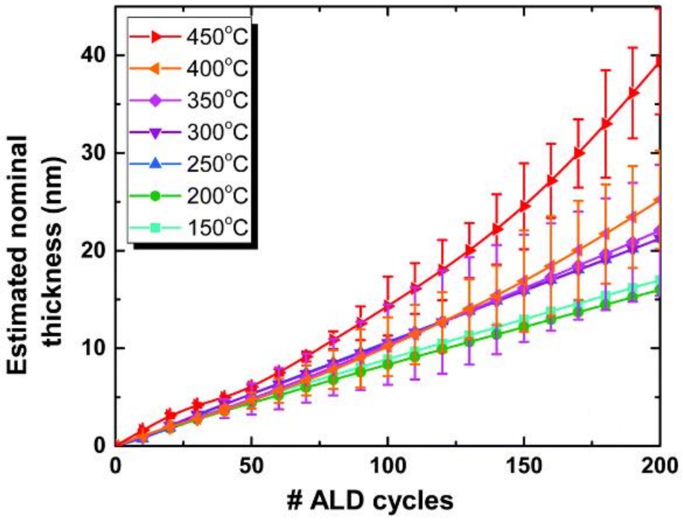


**Figure S20.** Layer thickness evolution as a function of number of ALD cycles over the temperature range of 150 °C - 450 °C. Reproduced from Ref. 1 with permission from The Royal Society of Chemistry.

**Table S11.** Summary of $C_{dl}$ obtained from fitting plots in **Figure S19**.

| Sample name | $C_{dl}$ (μF/cm$^2$) |
|---|---|
| 2H-$MoS_2$ | 3.4 |
| 2H-$MoS_2$-after *operando* XAS | 4.4 |
| 2H-$MoS_2$-after stability test | 3.9 |
| 1T-$MoS_2$ | 161.1 |
| 1T-$MoS_2$-after *operando* XAS | 108.9 |
| 1T-$MoS_2$-after stability test | 56.6 |
| Am-$MoS_2$ | 17.6 |
| Am-$MoS_2$-after *operando* XAS | 51.0 |
| Am-$MoS_2$-after stability test | 39.9 |

## Part VI: Density Functional Theory (DFT) modeling

Density Functional Theory (DFT) calculations were carried out using the Vienna ab-initio simulation (VASP) package.[7] The electronic exchange-correlation potential was described using the Perdew, Burke, and Ernzerhof (PBE) functional.[8] Van der Waals interactions were taken into account with the D3 method of Grimme *et al.* with Becke-Johnson damping (DFTD3(BJ)).[9] The kinetic wave functions were expanded in a plane wave basis with a high energy cut-off of 600 eV and the convergence criterion was set to $10^{-6}$ eV between two ionic steps for the self-consistency process. A monolayer of the (0001) basal plane was constructed by 3×3 unit cell of $MoS_2$ in the ***a*** and ***b*** directions with a vacuum region of 20 Å added along the normal ***c***-direction to avoid interactions between adjacent images. The Mo-terminated edge were created by truncating $MoS_2$ monolayer along the $(10\bar{1}0)$ crystallographic plane for

the calculation of the Gibbs free energy of hydrogen adsorption ($\Delta G_H$). The Brillouin zone was sampled using a 9 × 9 × 1 Monkhorst-Pack *k*-point mesh for both the basal (0001) plane and the (10$\bar{1}$0) Mo-edge. To investigate the effect of bond structures on hydrogen evolution activity, we have calculated Gibbs free energy of adsorbed hydrogen ($\Delta G_{H*}$) on $MoS_2$ basal (0001) plane and the (10$\bar{1}$0) Mo-edge with regular and shorter bond distances as follows: $\Delta G_{H*} = \Delta E_H + \Delta E_{ZPE} - T\Delta S_H \approx \Delta E_H + 0.24$; where $\Delta E_H$ is the adsorption energy of hydrogen which is defined as: $\Delta E_H = E_{MoS2+H} - E_{MoS2} - {}^1/_2\, E_{H2}$. Under standard conditions, the zero-point energy minus the entropic term, $\Delta E_{ZPE} - T\Delta S_H$ is 0.24 eV. $\Delta G_{H*}$ is considered as a suitable descriptor of HER activity for a wide variety of metal catalysts and for an optimal HER activity to be achieved, the value of $\Delta G_{H*}$ must be close to zero, indicating that the free energy of adsorbed H is close to that of the reactant or product.[10]

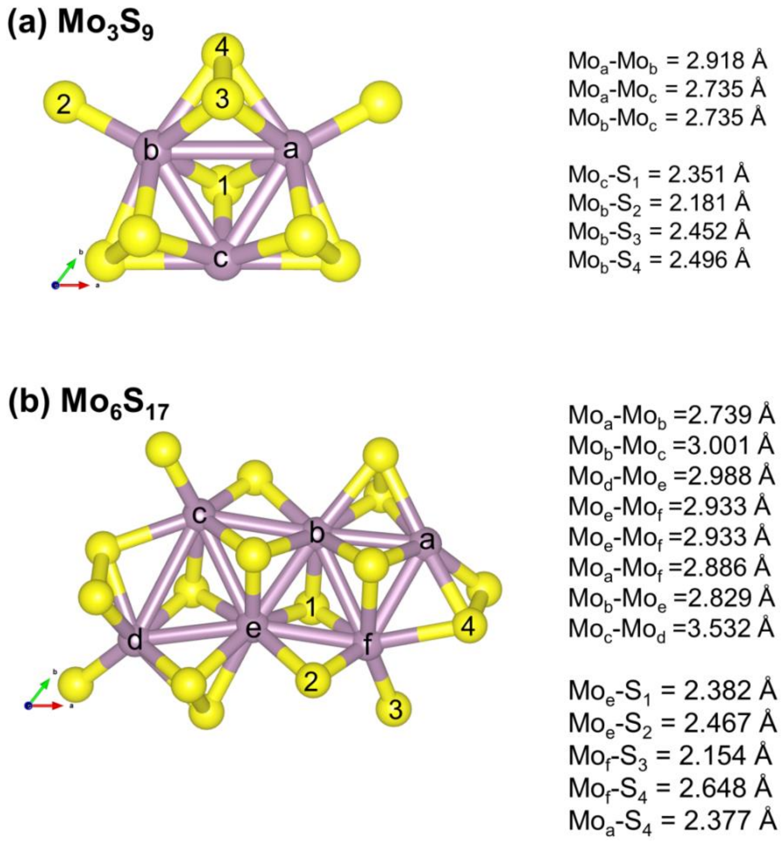


**Figure S21.** Optimized structures and calculated interatomic Mo−Mo and Mo−S bond distances in (a) $Mo_3S_6$ and (b) $Mo_6S_{17}$ clusters.

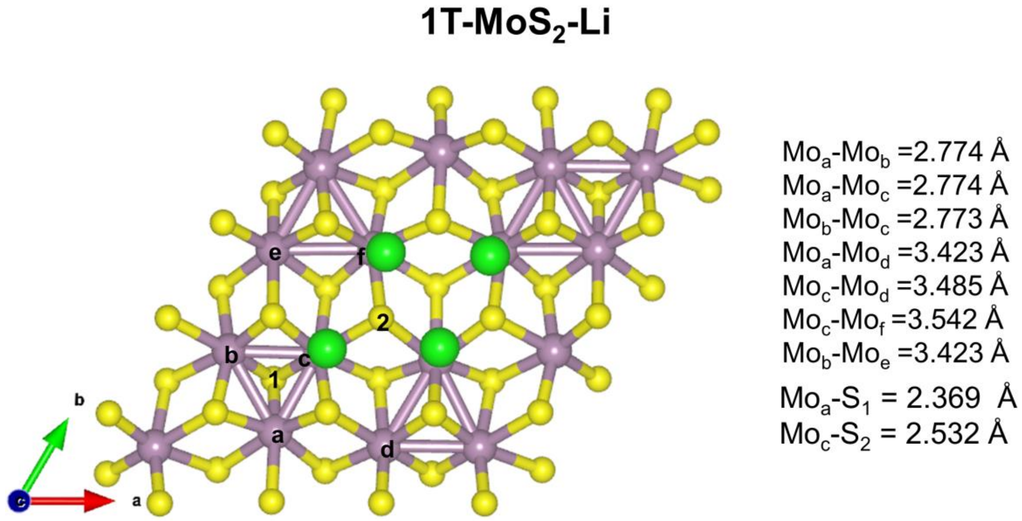


**Figure S22.** Optimized structure and calculated interatomic Mo−Mo and Mo−S bond distances in 1T-$MoS_2$.

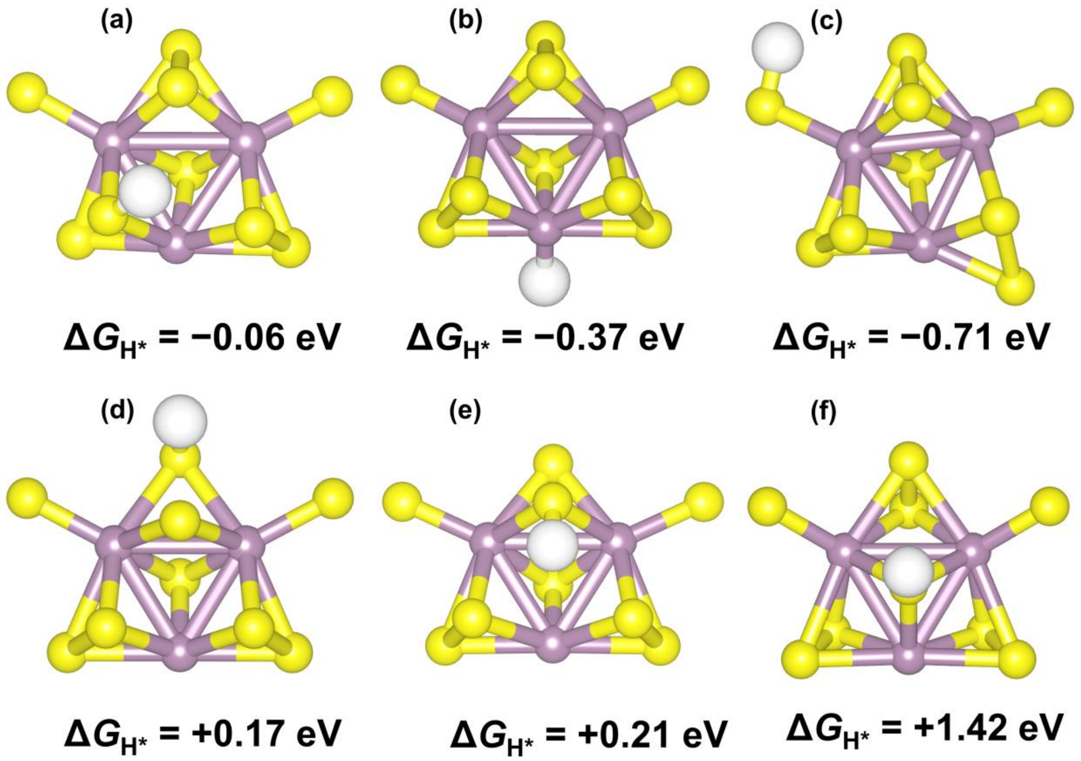


**Figure S23.** Optimized structure and calculated Gibbs free energy (eV) for hydrogen adsorption on $Mo_3S_9$ cluster.

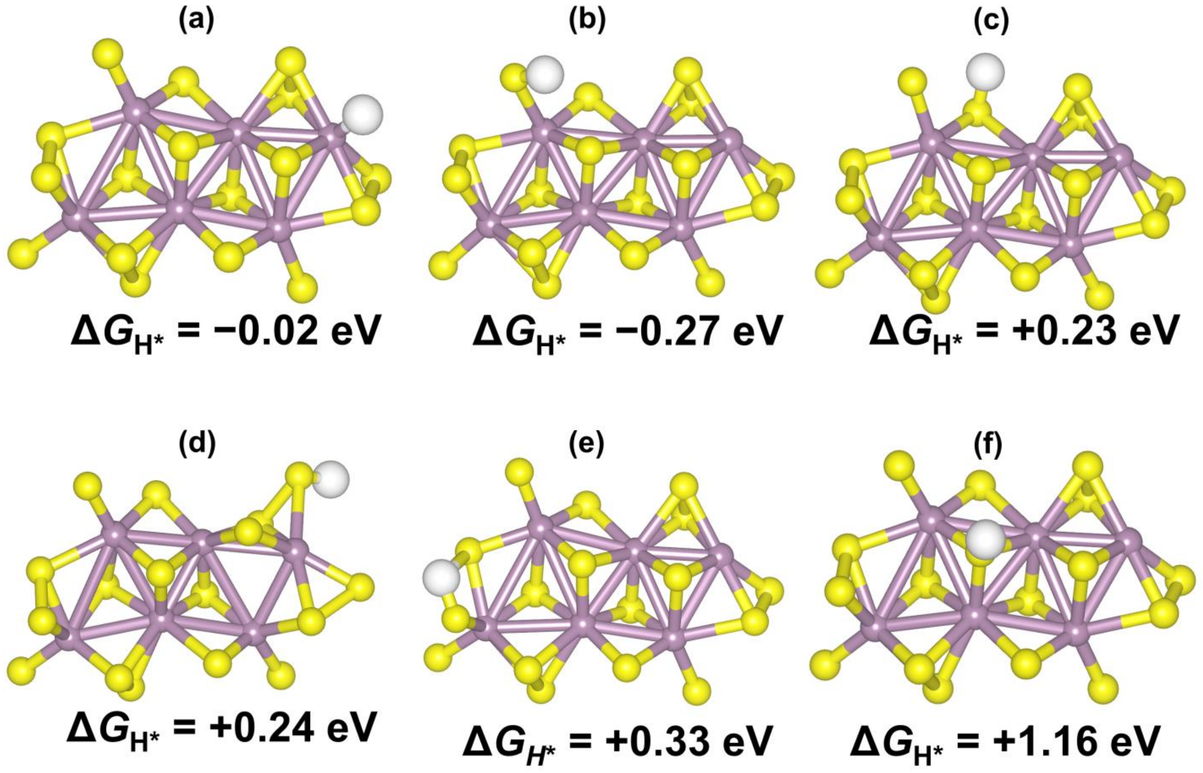


**Figure S24.** Optimized structure and calculated Gibbs free energy (eV) for hydrogen adsorption on $Mo_9S_{17}$ cluster.

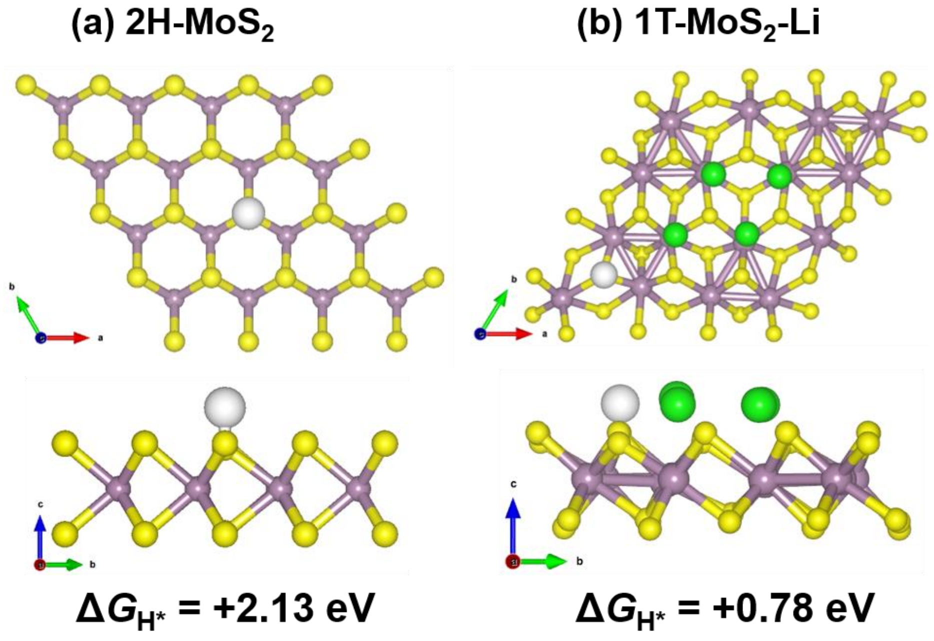


**Figure S25.** Optimized structure and calculated Gibbs free energy (eV) for hydrogen adsorption on (a) 2H-$MoS_2$, and (b) 1T-$MoS_2$.

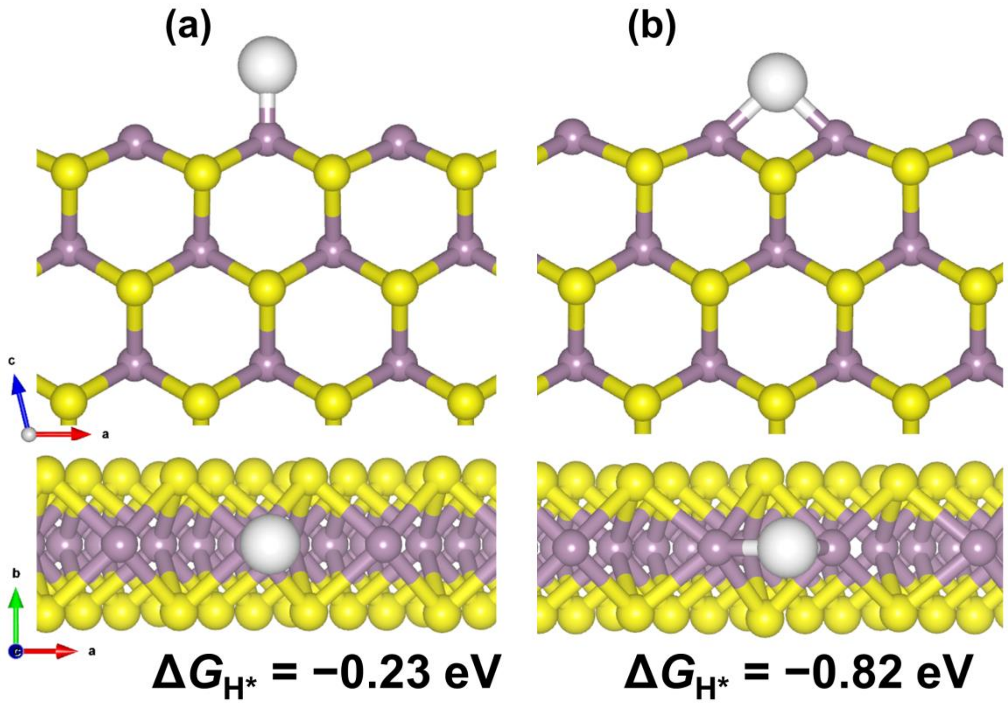


**Figure S26.** Optimized structure and calculated Gibbs free energy (eV) for hydrogen adsorption on 2H-$MoS_2$ Mo-edge.

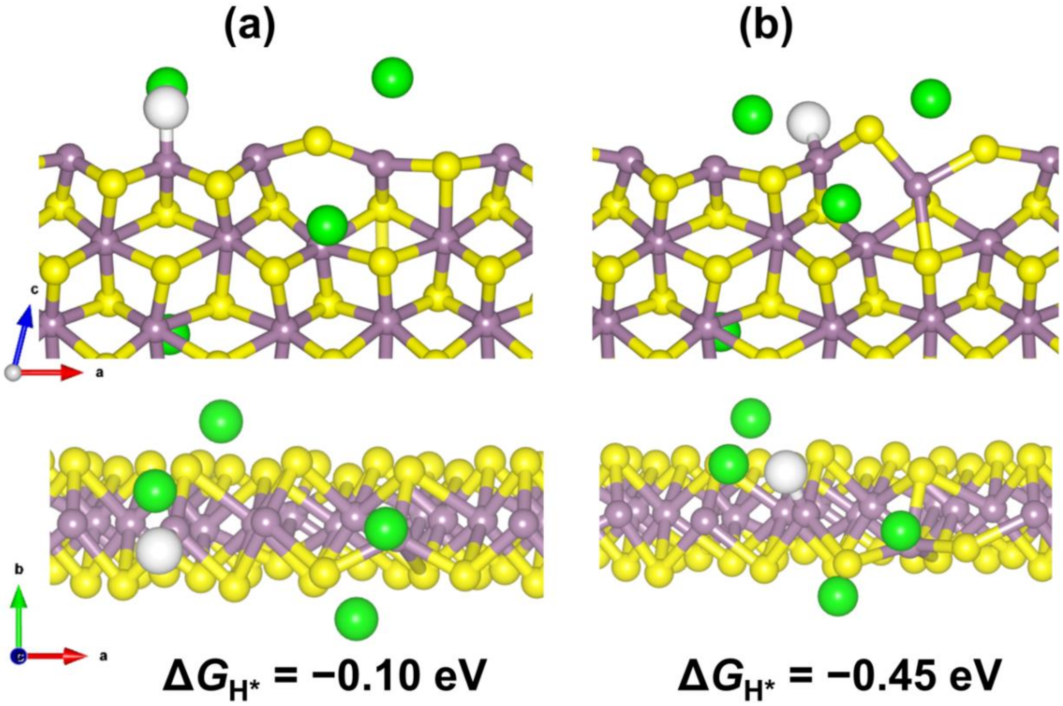


**Figure S27.** Optimized structure and calculated Gibbs free energy (eV) for hydrogen adsorption on 1T-$MoS_2$ Mo-edge.

**References**


[1] A. Sharma, M. A. Verheijen, L. Wu, S. Karwal, V. Vandalon, H. C. M. Knoops, R. S. Sundaram, J. P. Hofmann, W. M. M. Kessels, A. A. Bol, *Nanoscale* **2018**, *10*, 8615-8627.

[2] K. V. Klementev, *J. Phys. D: Appl. Phys.* **2001**, *34*, 209-217.

[3] a) B. Ravel, M. Newville, *J. Synchrotron Radiat.* **2005**, *12*, 537-541; b) R. T. Downs, M. Hall-Wallace, *Am. Mineral.* **2003**, *88*, 247-250.

[4] Y. Joly, *Phys. Rev. B* **2001**, *63*.

[5] S. J. R. Tan, S. Sarkar, X. Zhao, X. Luo, Y. Z. Luo, S. M. Poh, I. Abdelwahab, W. Zhou, T. Venkatesan, W. Chen, S. Y. Quek, K. P. Loh, *ACS Nano* **2018**, *12*, 5051-5058.

[6] S. Trasatti, O. A. Petrii, *Pure & Appl. Chem.* **1991**, *63*, 711-734.

[7] a) G. Kresse, Furthmüller, J., *J. Comput. Mat. Sci.* **1996**, *6*, 15-50; b) G. Kresse, J. Hafner, *Physical Review B* **1993**, *47*, 558-561.

[8] J. P. Perdew, K. Burke, M. Ernzerhof, *Phys. Rev. Lett.* **1996**, *77*, 3865-3868.

[9] S. Grimme, J. Antony, S. Ehrlich, H. Krieg, *J. Chem. Phys.* **2010**, *132*, 154104.

[10] a) J. K. Nørskov, T. Bligaard, A. Logadottir, J. R. Kitchin, J. G. Chen, S. Pandelov, U. Stimming, *J. Electrochem. Soc.* **2005**, *152*, J23-J26; b) J. Greeley, T. F. Jaramillo, J. Bonde, I. B. Chorkendorff, J. K. Norskov, *Nat. Mater.* **2006**, *5*, 909-913; c) P. F. Fewster, *Rep. Prog. Phys.* **1996**, *59*, 1339-1407.
